# Marcel Tanner, Global Health Specialist “Extraordinaire” Incl Supplementary Materials with Personal Contributions from Renowned Experts

**DOI:** 10.3390/diseases10040074

**Published:** 2022-09-30

**Authors:** Robert Bergquist, Lukas Meier

**Affiliations:** 1 Ingerod, SE-454 94 Brastad, Sweden; 2 Swiss Tropical and Public Health-Institute (Swiss TPH), University of Basel, 4123 Allschwil, Switzerland

Marcel Tanner, President of the Swiss Academy of the Sciences, Director emeritus of the Swiss Tropical and Public Health Institute (Swiss TPH), and Professor of Epidemiology and Medical Parasitology at the University of Basel, Switzerland, is indeed extraordinary, especially when considering the broad set of global health issues covered by his research. Trained as a parasitologist with a special focus on malaria and schistosomiasis, he skillfully combines various fields of expertise from basic to operational research; from molecular approaches to health systems; from infectious to non-communicable diseases; and from animal afflictions to human health (“One health”). Marcel’s contribution to science epitomizes the crucial iterative process, taking ideas from the laboratory to field implementation. He has always thrived when rapidly translating scientific findings into the real world to better the neglected populations and to improve underperforming health systems. He has strongly assisted scientific institutions in low- and middle-income countries and personifies the major drive behind the expansion of the Ifakara Health Institute (IHI) in Tanzania into a world-class organization for research, training, and services in the public health field. Centres in sub-Saharan Africa, such as the Centre Suisse de Recherches Scientifiques en Côte d’Ivoire (CSRS), the Centre de Support en Santé Internationale in Chad, and the Manhiça Health Research Centre in Mozambique, would not have become to what they are today without Marcel’s long-term support.

## 1. Key Interests

As a young scientist, Marcel took a keen interest the often complicated life cycles of parasites, carrying out research on human African trypanosomiasis, lymphatic filariasis, and schistosomes. Apart from cultivating filarial infectious stages [1], he proved that the antigenic variation in trypanosomes can be detected in the lymphatic nodes prior to reaching the blood stream, work that led to a new understanding of antigenic variation in vivo as well as new insights into why blood-stage trypanosomes can be cultivated in vitro [2].

Although biological mechanics are what caused Marcel to become interested parasitology in the first place, it led him to ongoing efforts to control endemic parasitic infections, which highlighted that any treatment would be complex. He soon concluded that vaccination should play the leading role in the same way that it does with respect to viral diseases. Travelling to Cameroon in 1979 together with his supervisor Professor Niklaus Weiss of the Swiss Tropical Institute (STI) to study onchocerciasis in the field, it dawned on him that the people they were treating suffered from much more than this one disease. Realizing that the overall situation largely also depended on other common afflictions of bacterial and viral origin, Marcel took an interest in working out control schemes based on a holistic approach that included consideration of the common lack of sanitation, nutrition, and access to reliable infrastructures. This was the base from which everything else followed through a shift from the initial, exclusive interest in biology to research on public health and, in due course, on health systems in general. Ready to move on in this direction, it was clear that knowledge, although valuable, also had to be applied. “You can never know everything, but you always know enough to do something”, he used to say. He also always voices his firm belief that “the joys in research are threefold: discovery, sharing and implementation”.

As author of over 800 original papers, Marcel was one of the six most productive scholars in health systems research between 1900 and 2012 [3]. However, he does not believe much in today’s many indices of personal impact, as they fail to provide a broad picture of progress, but rather feels that investment in the training of students at all levels by a variety of competent supervisors is more important. The reason for this is his belief that this approach not only helps individual careers but also paves the way for the development and application of new approaches while producing successful models of research partnership. Marcel has become an inspiration for new generations of scientists all over the world, opening up new vistas for the progress of health in the developing world. He was the principal investigator of the first field trial to assess the safety, immunogenicity, and protection of the synthetic malaria vaccine candidate SPf66 against the asexual Plasmodium falciparum blood stages [4]. Professor Tanner also led the trials for the RTS,S, the most advanced malaria vaccine candidate that has been developed so far, which is aimed at protecting young children against clinical *P. falciparum* malaria [5], which led to the announcement by the World Health Organization (WHO) on 6 October 2021 recommending widespread use of this malaria vaccine among children in sub-Saharan Africa and in other regions with moderate to high *P. falciparum* transmission.

Marcel Tanner has held key positions in global malaria initiatives. In 2010, he initiated a new series on malaria elimination in the *Malaria Journal* together with Professor Marcel Hommel. The series served as a guide to malaria elimination for decision-makers in malaria-endemic countries [6]. He was part of *MalEra* and *MalEra Refresh* (initiated by the Bill & Melinda Gates Foundation), putting forward a research and development agenda for the global elimination strategy [7]. He played a key role in the Malaria Elimination Group of the University of California. In 2016, the Director General of the WHO selected Marcel to head the WHO Strategic Advisory Group on Malaria Eradication (*SAGme*). The aim of the group was to evaluate whether global malaria elimination is scientifically feasible, and it concluded that eradication is a goal worth pursuing but also admitted that new tools are needed and that eradication will take time [8].

## 2. Neglected Diseases, Neglected Systems, Neglected Populations

The contextual determinants of health in endemic countries require a public health agenda that targets neglected populations and neglected diseases while also benefitting marginalized populations based on the principle of sustainability and a set of key determinants. The people who live in the developing world are not only financially marginalized, but also carry an inequitable part of communicable endemic diseases, which do not receive proper attention, as they are generally not subject to obligatory reporting. Today, we call these infections neglected tropical diseases (NTDs), as they are too often overlooked by the health sector despite the fact that some of them affect hundreds of millions of people. From the outside, they do not appear as the epidemiologic emergencies they are, which explains the lack of attention from the media. In addition, they are also mostly bypassed by the private sector, as they are not seen as worthwhile targets. Many of us working in this area know this all too well, and Marcel knows this better than anybody. In his words “We are not living in the first, second or third world—we are living in one world!”.

For Marcel, collaboration and research partnership is key. As director of the Swiss TPH, he has strongly promoted inter- and trans-disciplinary research, and his motto “If you want to solve a societal problem, it does not matter which discipline contributes the most—the solution is always trans-disciplinary” is a general principle that also affects approaches to disease control. Thus, it is not enough to produce and apply the necessary means; progress requires that the complete triangle of disease, health system, and population is applied in addition to long-term surveillance and response systems in order to achieve and sustain the elimination of an endemic, tropical disease. Indeed, Marcel was able to institute this kind of platform in China, which turned out to be an important adjunct to the elimination of the domestic transmission of filariasis and malaria in that country.

Emerging public private partnerships and product development partnerships epitomize a new form of collaboration between the pharmaceutical industry, academic institutions, and public/philanthropic donors, an area in which Marcel has played an important role by assisting the development of many virtual and real platforms. He was a founding member of the *Medicines for Malaria Venture* (MMV), chairing the *Drugs of Neglected Diseases Initiative* (DND*i*) and the Foundation for Innovative Diagnostics (FIND), and he was also a member of the scientific advisory board of the *Novartis Institute for Tropical Diseases* (NITD) in Singapore.

## 3. Health Systems: Thinking—And Acting

The organization of health systems reflects, as is common with institutional structures, both the history and the culture of the countries where they developed. Marcel has always sniped at provincialism and has looked for universal applications, and this is also the case here. One of his many contributions to the body of health systems concerns the consequent application of surveillance–response models for disease control. Furthermore, and based on the work in Chad, he and his partners were able to extend the “one medicine” approach to “One Health”, thus analysing human and animal health within ecological, social, and cultural systems [9]. Acknowledging the high merit of this work, the University of Zurich in 2020 honoured Marcel by making him doctor honoris causa.

The numerous short and longer contributions that are part of this editorial as well as the scientific papers in this Special Issue in honour of Marcel show the whole range of topics that he has initiated and developed throughout his life. They bear witness to his impressive publication activity and to the large network of partnerships and friendships that span the globe. They speak of a scientist who has nurtured and stimulated generations of students, who has built institutions, and who has stood up for integrity, but who also knows how to relax whenever and wherever the sun sinks—into the Kilombero River—or beyond. Contemplating the world on such occasions, he often utters the words “we are all in the same boat”, and these are not just empty words. We sincerely and warmly congratulate him on his 70th birthday and look forward to our further journey together.

## Supplementary Materials

The following are available online at https://www.mdpi.com/article/10.3390/diseases10040074/s1.
