# Supplementary material for: Marcel Tanner, Global Health Specialist “Extraordinaire” Incl Supplementary Materials with Personal Contributions from Renowned Experts
Source: Diseases. 2022 Sep 30;10(4):74. doi: 10.3390/diseases10040074 (PMC9590011; doi:10.3390/diseases10040074)
Supplement: Supplementary file 1 [file diseases-10-00074-s001.zip › diseases-1960964-supplementary.pdf]

diseases

Surveillance, pandemic preparedness, and the life and times of Marcel Tanner

Marcia C Castro [1,*] and Burton H. Singer [2,*]

[1] Department of Global Health and Population, Harvard TH Chan School of Public Health, 665 Huntington Avenue, Building 1, Room 1102A, Boston MA, 02115, USA; mcastro@hsph.harvard.edu
2 Emerging Pathogens Institute, University of Florida, Gainesville FL, 32611, USA; bhsinger@epi.ufl.edu
* Correspondence: MCC: mcastro@hsph.harvard.edu, BHS: bhsinger@epi.ufl.edu

1. On Marcel per se'

Having collectively known Marcel Tanner for 35+ years, it is difficult to reconcile the fact that he is now 70 years of age. Marcel has had a unique, productive, and highly innovative career, covering every aspect of tropical biology, epidemiology, and public health. He is also a skilled administrator, having developed the Swiss Tropical Institute from a small, limited topic group of 100 people to more than seven times that number plus paving the way for an elegant modern building as the home of what is now known as Swiss TPH (Swiss Tropical and Public Health Institute). Marcel also played a central role in expanding and upgrading the Ifakara Health Institute (IHI) in Tanzania and the Centre Suisse de Recherches Scientifiques (CSRS) en Côte d'Ivoire. There has more recently been the development of a new branch of the Ifakara Health Institute at Bagamoyo, emphasizing field work for vaccine development [1]. Moreover, Marcel has been active for many years in health systems development, a notable example of which was the Dar es Salaam Urban Health Project [2, 3].

A common theme in Marcel's health systems work has been surveillance as a critical tool for disease control and prevention. Surveillance, surveillance, surveillance!

Recent work focused on malaria eradication, quite naturally has surveillance as a key ingredient [4]. The Ebola outbreaks over the past ten years, vastly superseded by the SARS-CoV-2 pandemic have raised surveillance and, more broadly, pandemic preparedness, to a central focus in international health circles. The need for pandemic preparedness has been widely discussed in academic and health policy circles throughout the twentieth century [5, 6], but it has never received the international cooperative support that is needed to make it a reality. Marcel has already made some contributions to the pandemic preparedness literature [7]. However, the availability of new technologies in biological sciences and communications raises unprecedented possibilities for pandemic preparedness in the future.

We take this opportunity to present our views of where some of the most pressing issues in pandemic preparedness are situated looking ahead. One fully expects that Marcel will be an important leader in this expanding enterprise which will, of necessity, require an unprecedented international cooperative program. It will require the sustained support of governments, both national and local, around the world. If successful, global pandemic preparedness would be the most important activist international health program ever.

2. Why surveillance?

Public health surveillance is "the ongoing systematic collection, analysis, and interpretation of outcome-specific data for use in the planning, implementation, and evaluation of public health practice." [8]. It is the cornerstone for decision making – as Margaret Chan, the former Director-General of the World Health Organization, used to say: "what gets measured gets done." Without surveillance systems countries are limited in their ability to measure, monitor, and control ongoing health threats, evaluate and finetune policies, and detect new public health emergencies.

In the context of malaria elimination, surveillance is so critical that one of the three pillars of the Global Technical Strategy (GTS) for Malaria 2016–2030 is to transform malaria surveillance into a core intervention [9]. Marcel was and is a major voice spreading this concept. He emphasizes the regular collection of minimum essential data that can lead to action toward elimination. Here, data are often in the form of epidemiological data gathered through Health Information Systems. However, surveillance may involve data of different types and collected in many forms, as we detail next.

3. New technologies: metagenomic sequencing and phylodynamic/phylogeographic prediction

Identifying epidemics and pandemics of infectious diseases before they occur is a major challenge, particularly when the candidate pathogens are apriori unknown. Many epidemics and pandemics in the past [10] have involved organisms that had a known prehistory in wild animals before jumping to humans or even a prehistory of earlier outbreaks in humans — e.g., plague. Among the major challenges for the future are to reliably predict (i) which pathogens are most likely to engage in a species jump (wildlife to humans) and prove to be virulent in human hosts, and (ii) which pathogens among a group of them simultaneously infecting a given human have no prior record in the medical canon.

Before the recent availability of multi-genomic sequencing technology and phylodynamic methodology used for prediction, surveillance largely involved longitudinal assessments of humans and to a lesser extent, wildlife, with which they interacted. Repeated measurement was on viruses, bacteria, and parasites that were directly involved in human disease processes. The striking advantage of multi-genomic sequencing linked to phylodynamics is that cross-sectional (or repeated cross-sectional) data can suffice if the phylodynamic predictions are sufficiently trustworthy.

Beginning with Grenfell et al. [11], we have the initiation of combining meta-genomic assessments with a phylodynamic prediction for surveillance. This program has been greatly expanded over the past fifteen years, and whole new catalogs of previously unknown viruses are being evaluated as potential sources of future pandemics [12]. This strategy requires a set of cooperating labs and bioinformatics groups to effectively carry out the phylodynamics and interpret what it means. The larger problem in establishing wide-ranging capability in this domain is likely to be the availability of well-trained personnel rather than the availability of technical equipment.

4. Sero-surveys and their practical implementation

Sero-surveillance is important for indicating what has happened in the past. Here, a separate thread in surveillance strategies was initially put forth by Bryan Grenfell and colleagues [13] approximately twenty years ago. The basic idea was to establish a worldwide serology data bank, based on serosurveys at key sentinel sites. The conceptual development of this proposal has evolved considerably over the past two decades [14], and a multiplicity of serosurveys (some leveraging blood banks) have provided proof of principle that such a strategy could be an important contributor to a global pandemic surveillance system. Nevertheless, even in the era of COVID-19, getting to a global surveillance system with good prediction capabilities for new outbreaks and the appearance of new viral strains is far from becoming a reality.

5. The wildlife-human interface: from WHO to WOAH to integration of new technology with wildlife-human surveillance

A major shift in focus from disease reporting via the World Health Organization (WHO) to the World Organization for Animal Health (WOAH) will also be an essential part of a new surveillance infrastructure. Of considerable help is the fact that WOAH now reports diseases in wildlife, but these are already known pathogens and their consequences. Our more basic problem is the prediction of new pathogens in humans that are consequences of recent species jumps.

It is of critical importance to have global genomic surveillance and variant analysis capability if we are to ascertain the origin in animals of new pathogenic organisms. An important case in point is the recent investigation of COVID variants in South Africa [15].

Bat surveillance is important for the future [16] Educating the local public about disease risks is also critical. Then the question becomes one of maintaining public interest and supporting the surveillance. This connects with the monitoring of wet markets in Asia. A recent Opinion piece in the New York Times about the initiation and development of SARS-CoV-2 surveillance in the UK exemplifies the problem [17]. The technical capability is there, but the challenge must be sustained financing and with government support. How do you maintain long-term societal interest in a problem that is always present but that the general population is tired of hearing about it? It is interesting in this regard to review the experience of managing smallpox in the US 100 years ago [18].

6. Pandemic preparedness as such

Getting to pandemic preparedness has long been discussed [19], but whether via International Health Regulations or other more restrictive agreements (e.g., for Ebola) none of these more global schemes has succeeded in providing the level of international cooperation and technical support necessary for global pandemic preparedness to be a reality. Rather than discuss worldwide systems further, we think it useful to discuss preparedness within a single country that has a universal health system, with reasonably good data systems, and whose national surveillance could be adapted to widespread use of metagenomic/phylodynamic tools. Our focus for this discussion will be Brazil, where the government of Jair Bolsonaro has wreaked considerable havoc, during the COVID-19 pandemic, on a remarkably robust health system. However, the system was still strong enough to provide many positive examples of good functioning and to clarify how a national surveillance system for Brazil might practically be put into place.

Brazil is the only country with more than 100 million people that has a universal, free-of-charge, health system, which was created through social mobilization. The 1988 Brazilian Constitution guarantees health as a right to anyone and a duty of the state. The country

also has one of the largest community-based primary health care programs that has contributed to reducing health inequalities in access and mortality, and improving multiple health outcomes [20]. Regarding health data systems, Brazil routinely collects national data on births, deaths, hospitalizations, and immunizations, among others. Compared to other malaria-endemic countries, Brazil is in a much better position to use surveillance as a core intervention. However, at least three improvements are needed: (i) include a national ID number in all health systems to facilitate the linkage of data; (ii) implement routines of data science that quickly summarize and analyze data to support surveillance-response by local managers and public officials; and (iii) expand the digitalization of health units across the country to reduce the delay in data reporting.

As strong as health data systems can be, genomic surveillance is critical to monitoring the emergence of new pathogens, new variants of known pathogens, and the introduction/re-introduction of previously eliminated pathogens in certain areas. Currently, Brazil has three National Influenza Centers (NICs) that contribute to the WHO's Global Influenza Surveillance and Response System (GISRS) – they are in São Paulo, Rio de Janeiro, and Pará. In 2020, during the COVID-19 pandemic, Fiocruz Genomic Network was launched to study SARS-CoV-2 and its mutations, connecting 17 laboratories in 22 of the 27 states in the country. It is important to note that Brazil has, through its health system, a National System of Public Health Laboratories distributed in all states. In addition, several institutions and universities across the country have laboratories, some already part of national and international networks. Lastly, there is a vast network of private laboratories across the country. Here, Brazil is also in a good position to launch a nationally representative genomic surveillance network by leveraging, connecting, and expanding existing resources.

Expanding and supporting health data systems and genomic surveillance is feasible, but it requires a government willing to improve surveillance. As Marcel always says: "if there is a will, there is a way."

7. Corporate support for health services and disease surveillance: can this be expanded and maintained?

Extractive industries have a notable history of providing support for both health services and disease surveillance for limited periods when they first begin new facility construction. The philosophy has been that these companies are devoted to mineral extraction per se and that their health service forays are designed to provide start-up capability and training for local personnel that is subsequently to be taken over by the government of the country involved once production is initiated. The PNG/LNG liquid natural gas project is a notable case in point. Exxon/Mobile did a yeoman job with community health care that the PNG government could have well afforded to continue once the gas pipeline was operative. Regrettably, as in many similar instances in other localities, local political will was not focused on using corporate revenues paid during production to assist their people [21].

A still more dramatic case in point was the financing of schools, housing, roads, and health services in Chad during the construction phase of the Chad-Cameroon pipeline. Here, an autocratic political leadership in Chad directly inhibited continuing investments initially made by the Exxon/Mobil Corporation during the construction phase of the pipeline. Funds paid in from the corporate sector during production could have transformed Chad into a relatively wealthy modern country. The problem again was a total lack of interest

at the top of the government to facilitate any such economic and social transformation [22].

On the one hand, that corrupt governments may carry the programs into the future is one of the unfortunate features of the corporate investment experience. On the other hand, unsuccessful corporate support and the colonial nature of local businesses is quite problematic. Without some modifications in these experiences, the support of corporations toward worldwide pandemic preparedness is not likely to become a reality.

8. Marcel, looking ahead

Coming back to Marcel per se, some of his most obvious capabilities will be needed by the present and future leadership in health policy circles looking ahead. Three concrete examples can illustrate our point:

1. Marcel's well-known capacity for multi-tasking was on display from an early age. He and I (BHS) were both members of the Socioeconomic Research (SER) Steering Committee in WHO, Tropical Disease Research program (TDR) where we would be intensely reviewing and discussing projects for possible funding. While a given SER project was being debated, Marcel was critically evaluating a proposal for the Schistosomiasis Steering Committee, of which he was also a member. At decisive points in the SER review process, Marcel would look up, offer incisive and important commentary and recommendations, and, just as quickly, revert back to the Schistosomiasis proposal he was reviewing. During the course of any day, he would be evaluating a full complement of proposals for two steering committees simultaneously. Amazingly, the quality was always high while simultaneously exhibiting Marcel's ability to smoothly shift back and forth between disjoint topics over a period of eight hours.

2. Regardless of the topic you might raise pertaining to almost any aspect of tropical infectious diseases, control strategies, provision of health services, or even history and anthropology of a wide range of tropical localities, Marcel would always be one step ahead with explicit examples, references, names of people for you to contact if you want to press the subject further, and indications of relevant academic experiences within the University of Basel. Indeed, a new project would frequently be generated on the spot with protocols, people, and a timetable for completion. This was never a matter of one-upmanship. Marcel had already been wherever you were just now going and been there in great depth and detail. You often wondered whether Marcel Tanner was actually the name for a wide-ranging group of people rather than being a single individual.

3. Marcel's mentorship is one of the most important legacies of his career. He has inspired, trained, and mentored many (way too many to list here), in different continents and countries. Several of his mentees went on to become major scholars and leaders in the field. His generosity and humbleness were critical for this task. Anyone in the malaria field who does not know Marcel has not read and networked enough. Paving the way for the future generation of scholars is a trademark in Marcel's career.

Marcel usually says, "No roots, no fruits." His work and mentorship have established solid roots across many corners of the Earth, generated beautiful fruits that spread seeds and will continue to do so over generations.

References

1. Mwangoka. G.W.; Burgess, B.: Aebi, T.; et al. The Ifakara Health Institute's Bagamoyo Research and Training Centre: a well-established clinical trials site in Tanzania. *Int Health* **2009**, *1*, 85-90.
2. Harpham, T.; Few, R.; The Dar Es Salaam Urban Health Project, Tanzania: a multi-dimensional evaluation. *J Public Health Med*, **2002**, *24*, 112-119.
3. Harpham, T.; Tanner M. *Urban health in developing countries: progress and prospects*. Earthscan: New York, USA, 1995.
4. Rabinovich, R.N.; Drakeley, C.; Djimde, A.A.; et al. malERA: an updated research agenda for malaria elimination and eradication. *PLoS Med* **2017**,*14*, e1002456.
5. Carlin, E.P.; Graeden, E.; Robertson, H.; et al. Informing Pandemic Preparedness Through a Digital Global Health Security Library. *Health Secur* **2022**, *20*, 256-260.
6. Piret, J.; Boivin, G. Pandemics Throughout History. *Front Microbiol* **2020**, *11*, 631736.
7. Michel, J.; Sauter, T.C.; Tanner, M. What does a pandemic proof health system look like? *Glob Health Action* **2021**, *14*, 1927315.
8. Teutsch, S.M.; Churchill, R.E. *Principles and Practice of Public Health Surveillance*. Atlanta, GA, 1992.
9. The malERA Refresh Consultative Panel on Combination Interventions Modelling. malERA: An updated research agenda for combination interventions and modelling in malaria elimination and eradication. *PLoS Med* **2017**, *14*, e1002453.
10. Piret, J.; Boivin, G. Pandemics Throughout History. *Front in Microbiol* **2021**.
11. Grenfell, B.T.; Pybus, O.G.; Gog, J.R.; et al. Unifying the epidemiological and evolutionary dynamics of pathogens. *Science* **2004**, *303*, 327-332.
12. He, W.T.; Hou, X.; Zhao, J.; et al. Virome characterization of game animals in China reveals a spectrum of emerging pathogens. *Cell* **2022**, *185*, 1117-1129.
13. Metcalf, C.J.; Farrar, J.; Cutts, F.T.; et al. Use of serological surveys to generate key insights into the changing global landscape of infectious disease. *Lancet* **2016**, *388*, 728-730.
14. Mina, M.J.; Metcalf, C.J.; McDermott, A.B.; et al. A Global lmmunological Observatory to meet a time of pandemics. *Elife* **2020**, *9*.
15. Tegally, H.; Moir, M.; Everatt, J.; et al. Emergence of SARS-CoV-2 Omicron lineages BA.4 and BA.5 in South Africa. *Nat Med* **2022**.
16. Temmam, S.; Vongphayloth, K.; Baquero, E.; et al. Bat coronaviruses related to SARS-CoV-2 and infectious for human cells. *Nature* **2022**, *604*, 330-336.
17. Quammen, D. We are still in a race against the Coronavirus. New York Times. 2022 August 10, 2022.
18. Smallpox. *JAMA* **1922**, *79*, 304-305.
19. Gostin, L.O.; Katz, R. The International Health Regulations: The Governing Framework for Global Health Security. *Milbank Q* **2016**, *94*, 264-313.
20. Castro, M.C.; Massuda, A.; Almeida, G.; et al. Brazil's unified health system: the first 30 years and prospects for the future. *Lancet* **2019**, *394*, 345-356.
21. Castro, M.C.; Krieger, G.R.; Balge, M.Z.; et al. Examples of coupled human and environmental systems from the extractive industry and hydropower sector interfaces. *Proc Natl Acad Sci* **2016**, *113*, 14528-14535.
22. Polgreen, L. Chad backs out of pledge to use oil wealth to reduce poverty. New York Times, December 13, 2005.

From the Heart

Jacques Derek Charlwood[1]

[1] Global Health & Tropical Medicine (GHTM); jdcharlwood@gmail.com

With Marcel, basically, what you see is what you get. Of the other project leaders or Institute Directors that I have known, from many countries, Marcel is only one of two that I count as a friend. If ever I wanted a reference I would turn to Marcel and he always obliged. That must always have been a pretty hefty recommendation, because I often got the job. He is the only one who I would still travel abroad to visit (although, of course, I would combine it with visits to other friends and colleagues). Dinner at Marcel's, with Suzy Marcel's beautiful wife, was always the high point of a trip to Basle. Visiting the Swiss Tropical Institute, as the Institute was then called, would always feel like 'coming home'. Indeed, it was a characteristic of Marcel's that he managed to run a 'ship' in which all of the staff became friends. This may be a Swiss thing, but I think it was Marcel's doing. At the same time they did, and still do, cutting edge science.

The month or so that I actually spent working in Basle, during an otherwise protracted period of navel gazing, was one of the happiest and most productive times of my somewhat spotty career. I stayed in a hostel a bike ride from town. There was a full sized swimming pool in the basement which I used in the mornings; my days were spent working with Tom Smith on entomological data of the first malaria vaccine trial in Africa, the famous or infamous Patarroyo vaccine; and at night I was introduced to Alpha Blondy and "Guggemusik" by the two remaining 'witches' of an earlier coven. I became a surrogate 'witch' after the recent departure of the 'bona fide' one back to the UK, and fell in love with the other two members of the triumvirate.

Tom and I produced five, really good, papers in four weeks, I think. (This is probably because I kept my finger out of the fieldwork once it had been set up, thus making the data easier to analyse than the stuff I normally give him).

When the entomology course in Eritrea really looked like it was going to turn into a book I naturally asked Marcel to write the Foreword, which he did; although I still dispute his version of events!

When Marcel used to visit Ifakara people buzzed round him, always wanting something. There were queues, both of old friends from his days as the Director there, and staff (with all their work related questions). I used to make sure that time spent in my house was away from all that. Rather, discussions of Bob Dylan or Keith Jarrett, whilst lounging on a sofa with a drink of Scotch - a tipple that Marcel introduced me to. Many a fine whisky have I drunk since then!

We are all getting older and by the time this is published the World will have changed (I write in the middle of March 2022). One should make good use of the time that is left, including more Dylan and Jarrett!!

A Celebratory Ode to Marcel Tanner

Daniel G. Colley[1]

[1] Department of Microbiology, University of Georgia; dcolley@uga.edu

Born in Basel sometime in the past
Marcel Tanner was made strong to last

He never wavered in any of his goals
Down steep mountains or over fast shoals

His education came at home and the field
His "Tryps" first in culture, then filarids did yield

Some immune-parasite work he then did
The world quickly knew he was no longer a kid

He was off to Ifakara to be Head of a Lab
For his home STI the results were just fab

He learned a great deal of malaria and schisto
Then epidemiology he joined in the mix-o

For soon public health, both planning and doing
Was his new focus and what he soon was pursuing

Implementing studies and work on control
That's where our Marcel found his next role

Soon he was known 'round the world as a leader
At home and abroad as a first-class teacher

Many a program wanted his time
On world-class panels his time was sublime

The WHO would not leave him alone
It caused lots of travel, he could hardly get home

Yet he juggled it all for many, many years
Writing all those papers and meshing those gears

But he never forgot where his loyalties lie
He never bought into that "pie in the sky"

He's stayed committed to those in his work
All for the children and even the clerk

Being a leader requires many things
And we know together they're what Marcel brings

Some are easily seen, and others are not
And those who know him, know he's always on spot

For this Basel product has made a major impact
And he's done so globally with both poise and tact

Public health in the tropics owes him a great debt
And I have a feeling that he's not done yet

His time's been well spent helping those in the world
And always presenting STPHI's flag unfurled

But he's well earned some rest by training the best
And being the host to many a guest

I hope he will now have some time for himself
Perhaps he can even put his suitcase on a shelf

We now toast Marcel, for his legacy's been earned
So, let's thank him now for all that we've learned

Happy 70th Birthday, Marcel.
Best - As always, Dan

Marcel Tanner and innovative drug discovery for neglected tropical diseases

Thierry Diagana[1,*], Feng Gu, Ujjini Manjunatha, Srinivasa Rao, Hans Rietveld, Paul Herrling, Lutz Hegemann, Jonathan Spector[1,*]

[1] Novartis Institutes for Biomedical Research; thierrydiagana@novartis.com

Marcel Tanner's impact in parasitology and public health is renowned. Perhaps less well recognized, though cause for equal celebration, are his contributions to drug discovery for neglected tropical diseases. The art of bringing innovative new medicines from the laboratory bench to the bedside relies on integrated multi-disciplinary expertise across basic research, preclinical science, trials, patient care and global partnerships. For decades, Marcel has selflessly applied his inimitable experience across that continuum to advance the delivery of essential new therapies for underserved patients in global settings. These efforts poignantly illustrate Marcel's ideology for maximizing the practical implications of tropical disease research, and the result has been an indelible influence on a pipeline of innovative drug candidates at our labs and others that has potential to affect many millions of lives for years to come. Here we reflect on Marcel's exceptional support of R&D for neglected tropical diseases at Novartis.

1. Guiding the launch of R&D for NTDs

Less than 3 kilometers from the Swiss Tropical and Public Health Institute (Swiss TPH) in Basel are the headquarters of the global medicines company, Novartis. That adjacent physical proximity has mirrored the close collaborative ties that have existed between Marcel and the leadership and scientific teams at Novartis from the earliest days of its tropical disease R&D.

Spurred in part by the company's experience developing the first artemisinin-based combination therapy (Coartem®) for malaria, which was first approved in Gabon in 1998, Novartis partnered with the Singapore Economic Development Board (EDB) in 2002 to establish a comprehensive pharmaceutical research institute focused on neglected tropical diseases with the intention of delivering novel medicines to underserved patients at non-profit. Thus, the Novartis Institute for Tropical Diseases (NITD) was founded at the Biopolis, a state-of-the-art biomedical sciences hub in Queenstown, Singapore. The labs were outfitted and approximately 100 chemists, biologists, parasitologists, and other associates were hired over the next few years. The stage was set for pharmaceutical science dedicated to longstanding health challenges, but Novartis was faced with a conspicuous gap—at the time it had little internal experience in R&D for "global health" infections and other diseases that predominantly affected patients in tropical, low resource parts of the world.

Novartis sought the guidance of a handful of external specialists who could help to effectively link the lab work, scientifically and strategically, with the precise needs of this new target population. Enter Marcel, who was a natural fit for this type of collaboration since, in the words of one of our scientists, "Marcel is simply expert in everything related to tropical diseases." Marcel joined NITD's Scientific Advisory Board (SAB) and quickly became a confidant to both Novartis senior management as well as the NITD leadership and scientific teams.

Marcel was in good company on the inaugural NITD SAB, which also included two Nobel Prize laureates, Sydney Brenner & Rolf Zinkernagel. The dynamic of the SAB in those early years was described as "friendly, but with the weight of evaluation and a critical eye. After all, the stakes were high given the ambition to discover breakthrough treatments for severely neglected tropical conditions." The SAB was charged with identifying gaps in NITD's research plans and helping to solve them. Marcel was as dominant a figure in the SAB then as he is now. Twenty years later he is the longest standing member of the SAB, having never missed a year of meetings. Moreover, his contributions were never limited to interactions in the offices and labs—rather, Marcel personally introduced Novartis scientists to patients and health systems through field-based activities that were linked with research Symposia in countries where diseases of interest were highly endemic (described more below).

As one Novartis leader summarized, Marcel contributed a special ability to "repeatedly and consistently bring the right people together to make connections to advance drug discovery for neglected tropical diseases. In that spirit, Marcel was also an early adopter of cross-sector collaboration—he embraced this idea at a time when it was not a common practice and saw the potential for partnership with organizations in the private sector that were taking these issues seriously."

2. Advocating for malaria research

A few years after NITD was established, Marcel played an active role in helping to expand the scope of work to protozoan parasites. *Plasmodium* was the first pathogen to be addressed. A springboard for collaboration in this area was already in place given that Marcel had helped to guide the strategy for clinical development of Coartem,® working with Novartis' predecessors in CIBA-Geigy (Novartis was formed in 1996 through a merger involving Ciba-Geigy and Sandoz). The infrastructures that Marcel helped to assemble in Ifakara, Tanzania, were then involved in the registration trials for Coartem®. Later, to advance the development of a pediatric formulation in collaboration with Medicines for Malaria Venture (MMV), researchers at Ifakara worked with local schoolchildren to test palatability of different flavors and were integrally involved in the pivotal study of Coartem® Dispersible.

Marcel was aware of early signals from the field suggesting that improper marketing of artemisinin monotherapies could risk the eventual development of drug resistance, and he joined NITD leaders to advocate to Wellcome Trust for novel malaria drug research. "Marcel was among a group of concerned stakeholders who knew we needed to start working then to be prepared for a future where the efficacy of artemisinin-based combination therapies could potentially decline, and he was instrumental in helping to widen NITD's mandate to include malaria research," recalls a leader from Novartis' Malaria Initiative, "Marcel's activism helped to catalyze the first malaria research grant from Wellcome Trust to Novartis in 2007." This is how Thierry Diagana, who was recruited to lead the NITD malaria drug discovery program, met Marcel and worked closely with Marcel's team at Swiss TPH to deliver the first Proof-of-Concept in malaria in over two decades (Fig. 1).

The malaria program at NITD led to the discovery of KAE609 (cipargamin) and KAF156 (ganaplacide), drug candidates with new mechanisms of action that are currently in advanced clinical development. Furthermore, Marcel's connections (e.g., with the European and Developing Countries Clinical Trials Partnership) helped to facilitate the engagement of trial sites in countries where malaria is highly endemic. Other innovations in malaria

research at NITD include INE963, a potent and fast-acting novel chemical entity with potential for single-dose cure, and a state-of-the-art radical cure assay to support drug discovery against latent malaria infection caused by *Plasmodium vivax*—again, programs which have been bolstered by Marcel's insights and constant encouragement.

3. Expanding a focus on neglected parasites

The advent of precision medicine and targeted therapies over the last 10-20 years led to the discovery of important medicines to treat diseases across a range of disciplines. A natural effect was a growing emphasis on target-based drug discovery, to the extent that phenotypic screening was at risk of being considered comparatively suboptimal or inefficient. A Novartis leader remembers how "Marcel advocated for any tactic that kills the parasite! He was a champion for high throughput screening even if the mechanism of action was unknown. His view that we should not abandon this approach, which had served us well until then, was welcome reassurance and helped to keep us on track."

Marcel was of course intimately familiar with the unmet therapeutic needs in human African trypanosomiasis (HAT). He helped to educate Novartis scientists about the condition and, when the opportunity manifested to launch drug discovery efforts in this area, Marcel helped to facilitate links between Novartis, Swiss TPH, Drugs for Neglected Diseases *initiative* (DND*i*), and Wellcome Trust. The collaboration with Swiss TPH was crucial—Marcel brought Novartis scientists to the Swiss TPH labs and helped NITD to formulate a drug discovery strategy to address kinetoplastids. The result was a full screening campaign focused on HAT, with initial assays using *Trypanosoma brucei* strains that were shared by Swiss TPH, that then paved way for subsequent expansion of efforts to tackle *Trypanosoma cruzi*, the causative agent of Chagas disease.

4. Linking "bench" with "bedside" in global settings

Marcel came frequently to the NITD labs in Singapore ("he was right at home in that tropical environment," recalls a Novartis associate) and he helped to steward many field excursions. Marcel is remembered among the scientists as one of the Board members who most implored that the team spend time in countries where their target diseases were endemic to learn firsthand of the experiences of patients, health workers, and local researchers. Until that time, the scientists largely relied on published information and second-hand reporting. But the opportunity to visit clinics, experience health systems up close, understand the roles of local healers, and meet with local scientists had important implications for informing the target product profile (TPP) of new drugs and forging local collaborations—not to mention instilling an added sense of passion and urgency for the work taking place at the laboratory bench.

These experiences to better understand the "patient journey" were incorporated into research and educational Symposia that NITD regularly convened in countries where diseases of interest were endemic. Marcel was a fixture at these gatherings, which took place in Indonesia, Tanzania, Zambia, Cameroon, & Mozambique (Fig. 2). It became routine to extend the trips by several days to enable visits to villages and health care facilities, with Marcel always helping to organize those itineraries, provide context, and share his experiences. Moreover, for several years starting in 2007 NITD formed a research field station in collaboration with scientific and clinical partners in Jakarta and Makassar (Indonesia). One Novartis scientist reflected how "Marcel helped us to keep top of mind the fact that drug discovery is only one component in a complex set of needs where impact on patients is the most important ultimate outcome." This keen perspective, crucial to the goal of optimizing the potential of each medicine and helping patients at the bedside, was also cap-

tured in Marcel's didactic teaching through which he stressed the difference between efficacy of a medicine under controlled conditions (e.g., its performance in trials) and its effectiveness in real world settings.

5. Educating and inspiring drug discovery scientists

Many scientists at NITD have known and worked closely with Marcel for years. When asked about the personal impact Marcel has had on their programs and their own careers, it was challenging to capture the breadth of praises. "His energy is infectious. He is humble. He smiles all the time and is forever quick with a clever quip or anecdote." "His deep interest and commitment to neglected tropical diseases brushes off on everyone around him and guides us in the right direction." "Marcel always makes you feel like you are doing something important." "Marcel often made me nervous when we talked about details of our programs, and that is only because he is so insightful, and his perspective carries substantial weight with us. Working with Marcel always comes with growth and learning." "He listens. He has a curious mind. And he is constantly asking what he can do to help others around him." "As long as a problem was clearly spelled out and people were working on innovation to address it, then Marcel was supportive!" "Fundamentally, he is an optimist. He has a positive outlook on life. He feels there is hope and change can happen, and that is a message that is super encouraging for our young scientists." "People always remember the first time they meet Marcel—he is approachable, warm, supportive, and makes me laugh!"

The comment about Marcel's humor is worth highlighting. We recognize that Marcel's ability to provide such effective guidance to our teams stems in part from his natural manner of calling out issues or fallacies in ways that don't de-motivate but, rather, inspire and energize. Smiles and laughter are key ingredients. An illustration of this approach that some readers of this essay may recall is Marcel's use of a particular "donkey cart" parable in presentations (Fig. 3). An image captured from a village in Pakistan depicts an unfortunate (and bewildered) donkey fully lifted off the ground by a cart that has been loaded to the brim with various packets. The caretakers were left with two choices: pull on the reins to muscle the donkey back down to earth or lighten the load on the cart. This photo sparked many metaphors, including approaches to community health (e.g., how many tasks could villagers or community health workers be expected to perform?) and strategic program decisions (e.g., is it reasonable to take on new programs or is the portfolio already overloaded?). The poignant messages, always delivered with wit and sparkle, never failed to land and have stayed with us.

6. In summary: a durable legacy

Marcel's expert, passionate, patient-centric approach has contributed in palpable and enduring ways to R&D for neglected tropical diseases at Novartis. His singularly unique perspective and uncommon dexterity at establishing inter-disciplinary, cross-sector partnerships have led to outsized contributions, and we are grateful that he has chosen to partner with us for so many years. We know that privilege comes with responsibility, and we intend to consciously apply lessons from Marcel as we strive to embody the principles learned through the lived experience of working with him. We also acknowledge that Marcel's greatest impact as it relates to our work together may still be yet to come. As the drug candidates he has helped to shape progress through clinical development on their way to registration, Marcel's fingerprints will be on novel medicines for neglected tropical diseases that touch the lives of millions of families around the globe.

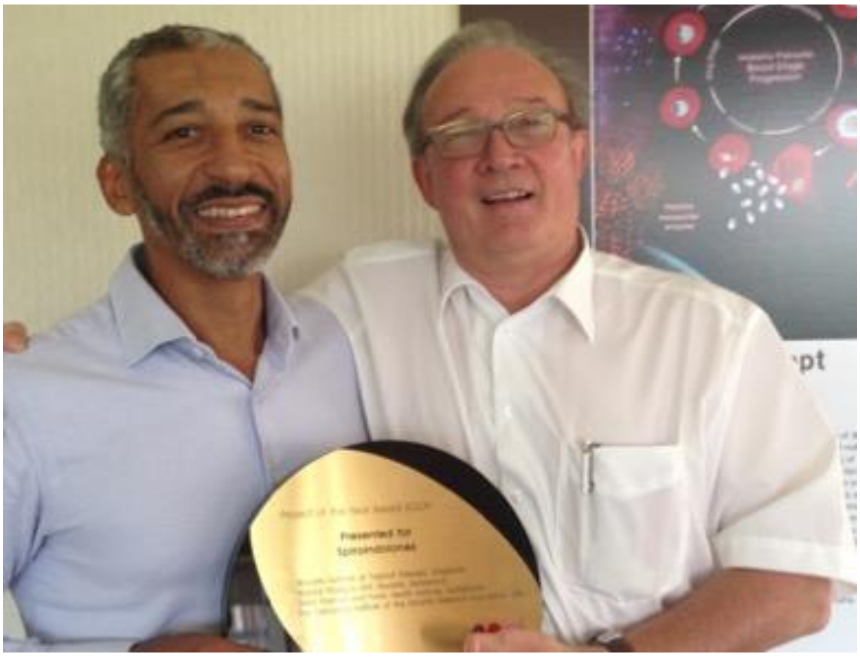

Figure 1. Recognition of the Proof-of-Concept trial for KAE609, a novel spiroindolone. Thierry Diagana and Marcel in Singapore sharing an accolade for the Medicines for Malaria Venture (MMV) "Project of the Year" award in 2009.

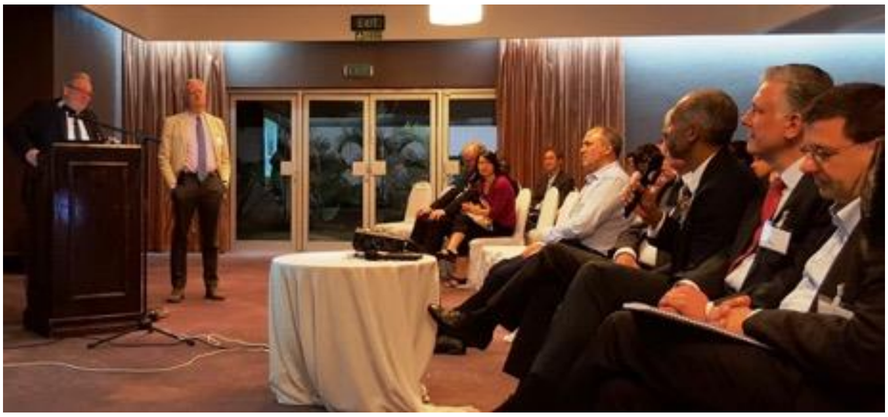

Figure 2. Marcel sharing insights with Novartis scientists and local researchers in Lusaka, Zambia, in 2016. Also pictured are Paul Herrling (former Head of R&D at Novartis; standing to the right of Marcel), Thierry Diagana (Head of NITD; with microphone in hand), and Lutz Hegemann (Head of Novartis Global Health; sitting next to Thierry).

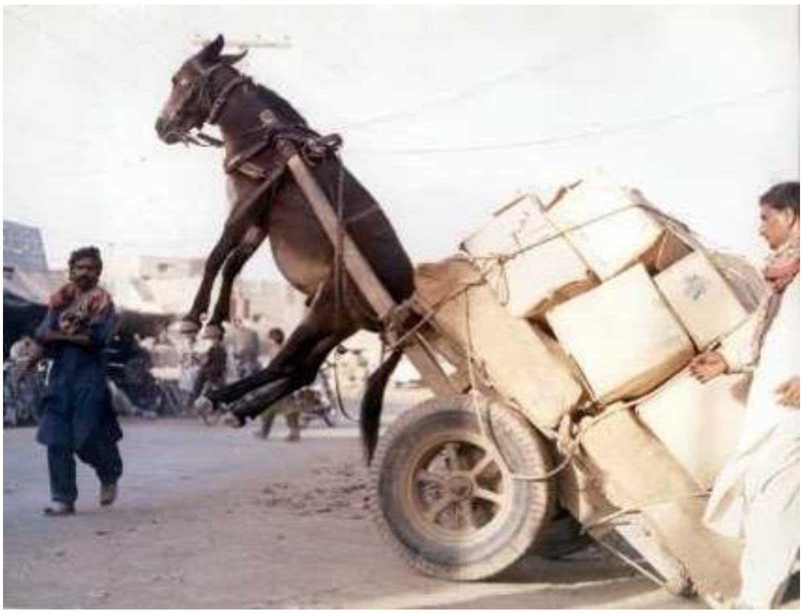

Figure 3. A curious spot to be in.

Recalling Marcel Tanner

Sharon Fonn[1]

[1] School of Public Health, University of the Witwatersrand, Johannesburg; sharon.fonn@wits.ac.za

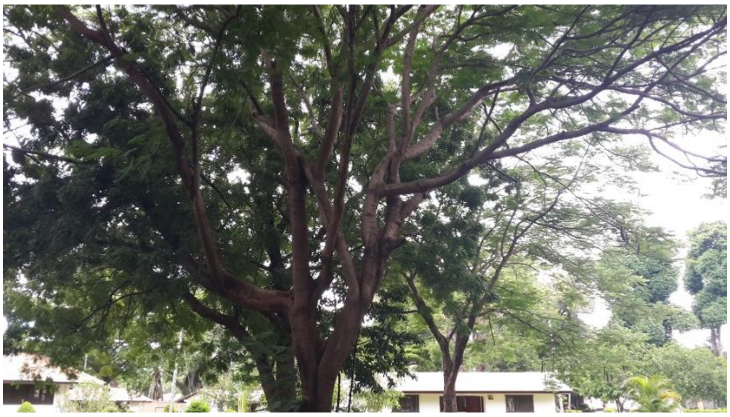

Figure 1. Campus, Ifakara Health Institute, Ifakara, Tanzania.

This may seem like a strange photograph to share when one is asked to recall Marcel Tanner. The foreground is a huge tree and only just visible two of many houses that make up the Ifakara Health Institute (IHI) campus in Ifakara, Tanzania. It is however fitting, trees take root and grow large over time. The campus too is a very solid, important, concrete, in the physical and metaphorical sense – one of the legacies that Marcel has left. I took this photograph when I attended one of IHI's strategic planning meetings in maybe 2015 as a member of their scientific advisory board. I reveled in the dynamic discussions held. I noted that Marcel may have spoken too much or maybe just enough as he said things others may think but not say. I reveled too in the humid gentle heat, the dusty wet smell that is Africa and that every African knows in their heart. Africa still seeks to make and claim its place in the world in general and the world of science and research in particular. There are many reasons for Africa achieving below its potential, and those of us who live here must call our governments to account. But there are structural inequalities as well that have a long history that have systematically underdeveloped Africa. Slave, missionary and colonial capitalist history must be told, owned and many suggest should result in the payment of reparations [1] . Marcel knew this and it was in his actions that I saw how scientists from the so-called global north could act with integrity. It is strange how so many still don't but that is an issue for another day.

Marcel, when I met him in the early 1990's, was clear that no Swiss post graduate student would get an opportunity for a degree in Ifakara without a Tanzanian counterpart having the same opportunity. Many Tanzanian masters and PhD students graduated because of that approach. IHI is owned by Tanzania and run by Africans because of Marcel's approach. Marcel has a huge intellect and vision and even a huge ego but an ego of a particular type. He shared his ideas, his networks, his contacts, his opportunities so that others too could grow and lead. He used his power for the benefit of others. He has used science in the service of increasing equity.

Today some may think that mixed methods is de rigueur, commonplace, almost necessary in public health research. This was not always the case however. In 1994 Marcel supported a qualitative methods workshop which was held in Ifakara [2]. The workshop brought

African students/researchers/practitioners with non-social-science backgrounds together for 3 weeks and qualitative methods were shared and applied within the community and nearby. This made an important contribution to what is now a well-known accepted method of research in public health. It was not Marcel's work alone, if I recall correctly it was the WHO Special Programme for Research and Training in Tropical Diseases (TDR) that facilitated the workshop. Marcel supported the work of the TDR Gender Task Force when we applied the approach of Paulo Freire to health systems research [3] at a time when neither were popular. Health Workers for Change [4] is one product of the TDR Gender Task Force – a curriculum that is still used today more than 20 years after it was developed and tested [5,6]. Marcel helped give legitimacy to things (methods, people, places, issues) that are of value but not always valued.

Others will talk of Marcel in relation to health and demographic surveillance sites (HDSS) and INDEPTH. I want to talk about its role in developing public health teaching in Africa. It was INDEPTH's understanding of the need to develop African researchers in Africa that led to the development of a Masters in Field-based Epidemiology at the School of Public Health at the University of the Witwatersrand, Johannesburg (Wits). Later this developed into a further area of academic qualification as we sought to create an academic field for data managers when we started another masters specializing into what is now data science. Fundamental to the success of both these programs was the combination of a taught component at Wits and a 6 month student placement at a range of the HDDS sites, including Ifakara, where the students did their masters research [7,8]. This approach also brought together universities and independent African research centers which was and is beneficial to both sets of institutions. Much of that experience was brought to bear when we developed the Consortium for Advanced Research Training in Africa. Over 200 Africans have enrolled to do their PhDs in public and population health through CARTA [9,10] and more than 130 have already graduated. Marcel may not have been present in every meeting or part of every decision as we developed these various consortia and research training programs. But he certainly planted the seed from which has sprouted this strong African tree and it is still growing.

References

1. Gosh, J. The journey to greater equality. *Lancet* **2022,** *400,* 18-19.
2. https://apps.who.int/iris/bitstream/handle/10665/60386/TDR_SER_RP_94.2.pdf?sequence=1&isAllowed=y. (Accessed on 05.09.2022).
3. Onyango, O. W.; Laisser, R.; Mbilima, M.; et al. An evaluation of Health Workers for Change in seven settings: a useful management and health system development tool. *Health Policy Plan* **2001**, *16(Suppl.1),* 24-32.
4. UNDP/World Bank/WHO Special Programme for Research and Training in Tropical Diseases. Health Workers for Change: a manual to improve quality of care, **1995**, World Health Organization.
5. Ratcliffe, H. L.; Sando, D.; Lyatuu, G.W.; et al. Mitigating disrespect and abuse during childbirth in Tanzania: An exploratory study of the effects of two facility-based interventions in a large public hospital. *Reprod Health* **2016**, *13*, 1-13.
6. Webber, G.; Chirangi, B.; Magatti, N. Promoting respectful maternity care in rural Tanzania: Nurses' experiences of the "Health Workers for Change" program. *BMC Health Serv Res* **2018**, *18*, 1-6.
7. Kellerman, R.; Klipstein-Grobusch, K.; Weiner, R.; et al. Investing in African research training institutions creates sustainable capacity for Africa: the case of the University of the Witwatersrand School of Public Health Masters programme in epidemiology and biostatistics. *Health Res Policy Syst* **2012**, *10,* 1-7.
8. Klipstein-Grobusch, K.; Chirwa, T.; Fonn, S. Current status and future prospects of epidemiology and public health training and research in the WHO African region. *Int J Epidemiol* **2013**, *42*, 1522-1523.
9. Ezeh, A. C.; Izugbara, C. O.; Kabiru, C.; et al. Building capacity for public and population health research in Africa: the consortium for advanced research training in Africa (CARTA) model. *Glob Health Action* **2010**, *3*, 5693.

10. Igumbor, J.O.; Bosire, E.N.; Basera, T.J.; et al. CARTA fellows' scientific contribution to the African public and population health research agenda (2011 to 2018). *BMC Public Health* **2020**, *20*, 1030.

Contribution de Marcel Tanner à la transformation du CSRS et à la recherche scientifique en partenariat

Olivier Girardin[1]

[1] Fondation rurale interjurassienne, Courtemelon; olivier.girardin@frij.ch

Marcel Tanner a contribué de manière déterminante à la transformation du Centre Suisse de Recherches Scientifiques en Côte d'Ivoire (CSRS) en un centre de recherche reconnu en Côte d'Ivoire et en Afrique. Il a inspiré plusieurs générations de chercheurs et a su leur communiqué sa passion pour la recherche en partenariat, orientée sur la production de résultats pour améliorer les conditions de vie des populations en milieu urbain et dans les zones rurales. Il a servi de catalyseur pour de nombreuses équipes, il a su mobiliser les énergies et la passion des jeunes chercheurs en leur montrant la voie et en leur offrant des perspectives. Il a toujours su allier d'une part la recherche de terrain, aux analyses en laboratoire et d'autre part la recherche, à la formation et à l'action, en faisant preuve d'une vision et d'une grande générosité. Il a toujours été disponible pour répondre aux questions des chercheurs juniors ou seniors, en les soutenant dans leur quête. Son sens de la bonne formule et des mots justes a permis de stimuler, inspirer et maintenir la flamme de nombreuses équipes, pour une recherche de qualité avec toujours en ligne de mire l'amélioration des conditions de vies des bénéficiaires. Les visites sur le terrain avec Marcel Tanner ont toujours été stimulantes et inspirantes, ses qualités humaines et son sens du contact avec les populations est remarquable, que ce soit avec les enfants, leurs parents, les familles ou les autorités locales. Il n'a jamais perdu de vue l'objectif final et il a trouvé les voies et moyens pour l'atteindre, en faisant preuve d'une créativité sans limite et d'une vision stratégique exceptionnelle. Marcel Tanner a su partager sa vision pour la transformation du CSRS : d'une station de recherche en un centre reconnu en Afrique et au niveau international. Il a entraîné dans son sillage étudiants, doctorants, chercheurs, professeurs, directeurs et ministres et il a su les enthousiasmer et les mobiliser pour cette belle aventure humaine.

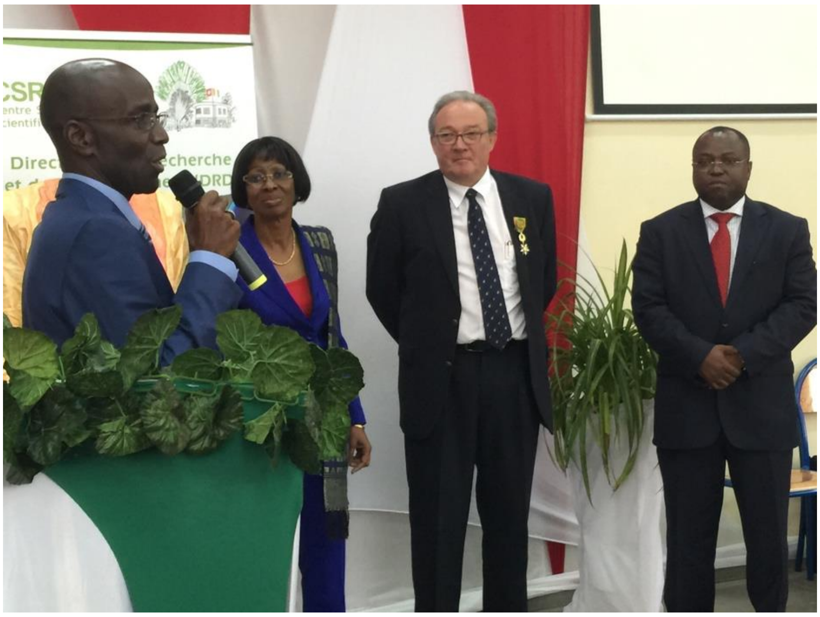

Figure 1. Marcel Tanner receiving the «medaille commandeur» by the government of Côte d'Ivoire.

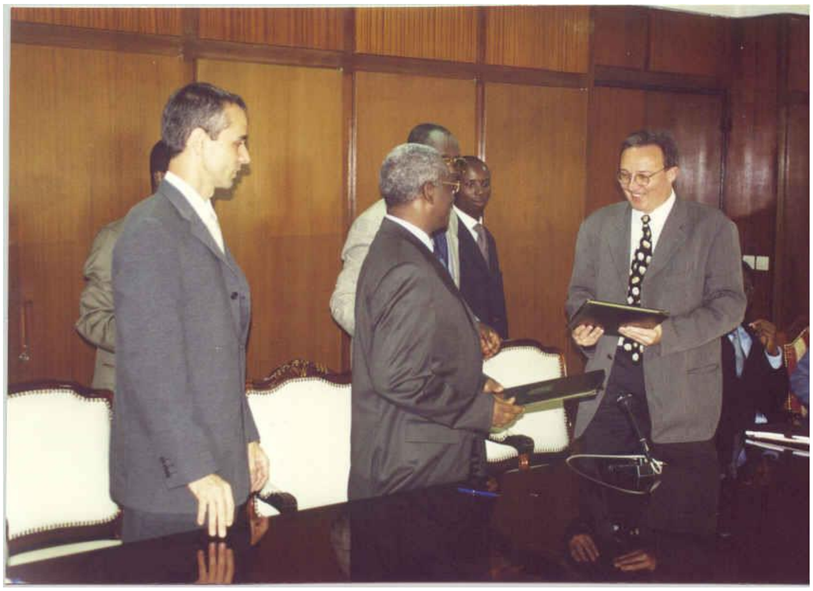

Figure 2. Marcel Tanner signing the "accord de siège" of CSRS.

All in the same boat

Christoph Hatz[1]

[1] Swiss Tropical and Public Health Institute (Swiss TPH), Switzerland; christoph.hatz@swisstph.ch

Dear Marcel
'Tupo pamoja': this was the case on June 30, 2015, at your official farewell from the Institute, and ever before and after, where it was and is important. 'Together in one boat' (Fig. 1) is one of your guiding principles, which has accompanied and supported us all in countless projects and activities of professional and private paths. And not only that: what has always united us and given us confidence is another basic idea, that in principle every idea gets the chance to be tested for its suitability. This has made it possible to innovatively tackle and realize many new projects that would otherwise have failed due to premature doubts or would have got stuck in the thicket of bureaucracy. The fact that not everything could be successfully implemented hurts less in this way. You always acknowledge and honor real greatness (Fig. 2). Well, the hunt for ngiri with Ambros Mganda, and the preference for cigars and fermented fruit, I do not necessarily have to share to understand and appreciate your enthusiasm and commitment in all relevant things. 'Nit lugg loh gwünnt' and mutual trust have brought the staff of the 'Tropeli' in general and for Ifakara in particular forward with united forces (Fig. 3). Young and old people have understood and internalized what matters for you: to work scientifically without compromise and to recognize and promote the interpersonal skills of each and every individual, this is the magic mixture with which things are achievable that cannot be conceived and implemented either at the square table or at the computer alone (Fig. 4). It continues as long as you have the desire and the energy, and as long as water flows down the Kilombero river (Fig. 5). There is fortunately still much to be tackled at local, national and international levels.

Thank you for everything, I wish you the necessary strength and health for the further plans.
Härzligscht,
Christoph

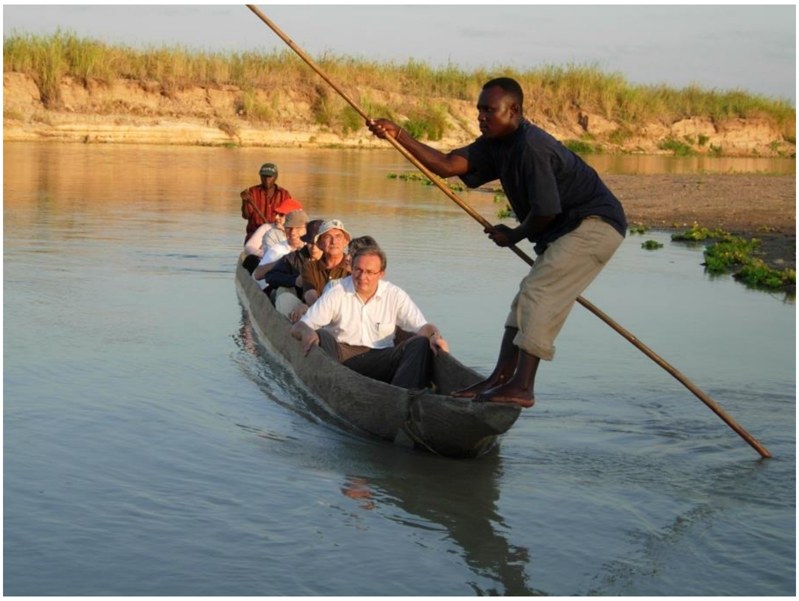

Figure 1. In the same boat on Kilombero river, Ifakara Tanzania.

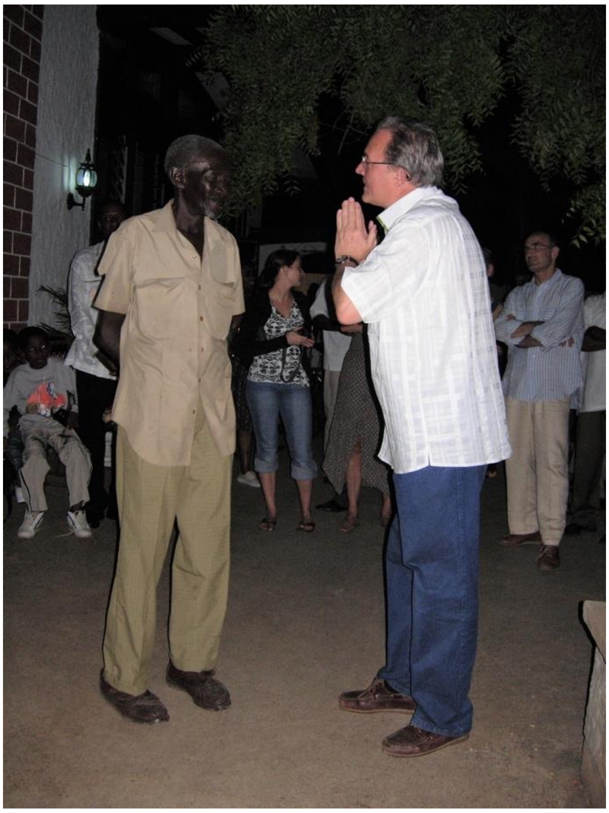

Figure 2. Mzee Ambros Mganda and Marcel Tanner.

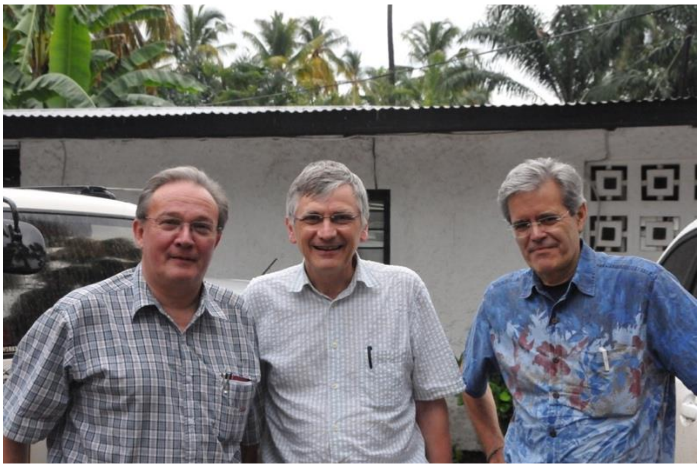

Figure 3. Marcel Tanner, Christoph Hatz and Don de Savigny (from left to right)

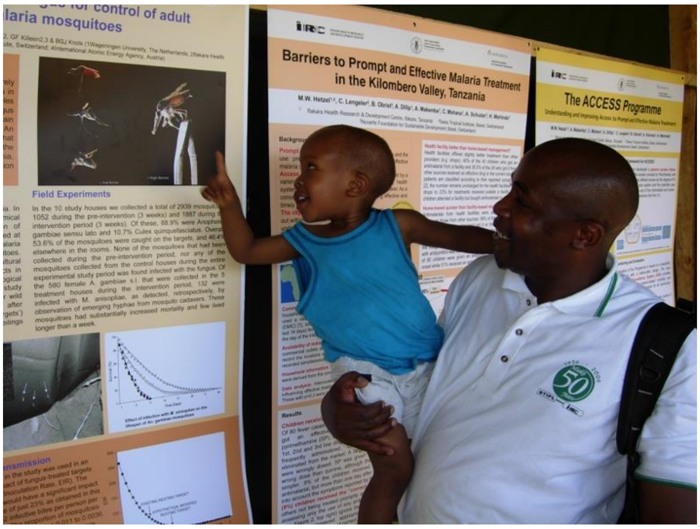

Figure 4.

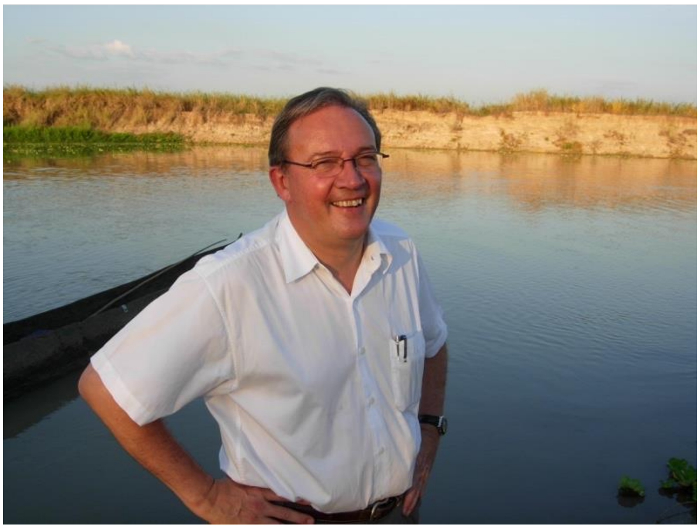

Figure 5.

Marcel Tanner: Some anecdotes

Paul Herrling[1]

[1] Former Head Corporate Research, Novartis, Switzerland; paulherrling@aol.com

Marcel and I started collaborating around 2001 at the time when Novartis was considering an increased investment in R+D for neglected diseases and patients and we made the decision to establish the Novartis Institute for Tropical Diseases in Singapore focusing on Dengue, Tuberculosis and later Malaria as resistances to artemisins were becoming probable. Marcel was an invaluable help as he had such an extensive experience of the public health aspects of these diseases and most importantly the environment and cultures of the patients affected. This was an area we had everything to learn having so far mostly been working in making medicines for the more affluent. Marcel joined us on our Scientific Advisory Board and by organizing scientific meetings and visits to hospitals in endemic areas in Africa and South East Asia he introduced me and the NITD scientists to the differences of making medicines for the affluent or for neglected patients in poorer regions of the world. The importance to understand the different cultural attitudes towards medicines as exemplified by the role of traditional medicine in South East Asia, the fact that clinical diagnosis of a patient there is publicly discussed in their community and in Africa the role of traditional healers and village elders amongst other aspects.

1. Demographic surveillance zones

One clever way to survey demographics including prevalent diseases are so-called demographic surveillance zones in the regions of the diseases of interest. The ones established by Marcel and his team around the research station of Ifakara in Tanzania worked by recruiting local young people from the villages to go to at regular intervals to every single habitation in a region. This was greatly helped by the fact that in these rural areas the government had allocated to each family a patch of land and a small dwelling, each with a unique number. The surveyors were trusted by the local population as they were part of it and would note the number of family members, births and deaths as well as diseases occurring in each family and other aspects of interest. Over time this resulted in eminently valuable data allowing an efficient allocation of health resources in a region and the same surveyors would bring the appropriate medicines to the families or arrange a trip to hospitals if required. One of the rather unexpected anecdotes around Marcel's demographic surveillance zones was due to the fact that one of the diseases monitored in the region was rabies. This disease was relatively frequent because of the many dogs in the villages. After one such survey it emerged that a few villages located relatively near to each other suddenly showed a much reduced number of rabies cases. As this result was repeated in subsequent visits an investigation was made with a rather interesting outcome: the Tanzanian government had decided to test a new, higher yield rice variety and had engaged in a collaboration with Korea to develop Tanzanian farmland, including the mentioned rice. Korea sent a number of Korean farmers to Tanzania to many villages including those in the demographic surveillance zone to plant it the rice and teach the local farmers how to grow it. --- It is the custom in parts of Korea to eat dogs as a delicacy, so the Korean farmers killed and ate the dogs in the villages they were… less dogs, less rabies… and shows how culture affects disease distribution.

2. Safari

A second anecdote with Marcel I wish to share is when again in during a time in Ifakara with Marcel I had a few days extra time he invited me to join him on a safari, but this was not a luxury lodge safari but one with his old Rover trackers from the Ifakara hospital and it entailed much walking to look for animals which we did plentiful. It was my first African safari and I really was fascinated and it motivated me to make several more. But this one was special for another reason. We spent the night in the bush sleeping on the ground around a huge fire intended to keep hyenas and lions at a distance which we could hear around us. We had a great dinner and Marcel cooked his traditional dessert of 'bananes flambees au brandy', after which we all had a good dram of malt whiskey and retired to sleep. I knew there where mosquitoes in this region so sprayed face and hands with repellent and slept with my boots on. I had forgotten mosquitoes can bite through some textiles, in this case my socks, so I woke up in the morning with two painful red rings of mosquito bites on my lower legs between the upper edge of my boots and the lower of the trousers… Exactly one week later back home on a Saturday I started feeling terrible, with rapidly increasing fever and headache so I called Marcel and described my symptoms. He said it was Saturday, no point going to hospital and he asked if I had coartem, a malaria medication Novartis had developed with Chinese scientists, and if I had I should take it immediately and if after a while the symptoms disappeared it was very likely malaria. So I started the treatment while frequently measuring my fever and writing down my symptoms. The fever peaked at around 41C during that night and continued unabated until next night when rather abruptly it started falling and I felt distinctly better… So the tropical medicines doctors agreed it was very probably malaria although a test for antibodies to *Plasmodium falciparum* was negative, probably because the medicine killed the parasite before antibodies were raised. So this trip with Marcel also gave me the very valuable experience to feel what the patients we were working for at NITD experienced… a very disagreeable and potentially dangerous disease.

I hope Marcel will continue for many years to share his immense know-how of tropical diseases and patients in the field with many more new workers joining the effort to alleviate the burden to neglected patients

When your boss always says YES!

Christian Lengeler[1]

[1] Swiss Tropical and Public Health Institute (Swiss TPH), Switzerland; christian.lengeler@swisstph.ch

By all accounts, for most Institute Directors in the academic world and beyond, the default answer when asked something involving additional resources is NO. For obvious reasons, it is the safe and prudent position to take, until evidence can be brought forward that the request is justified, can be financed, will bring increased visibility and fame, or fulfills whatever other criteria that makes it look like a worthwhile investment. With Marcel Tanner, his default answer was almost always YES. This is highly unusual, refreshing and very enabling for young scientists. His attitude contributed in a major way to the outstanding spirit of the Swiss TPH over the past three decades.

It takes a particular mindset as an institute Director to implement consistently the "yes by default" strategy. It certainly requires a positive and optimistic character, traits fitting Marcel Tanner as a person and as a scientist. He was a model for all of us in being constantly looking for solutions rather than lamenting about "problems". For always taking a positive perspective, and ultimately seeing everywhere potential for personal and institutional developments. For taking an acute interest in people, their plans and ideas, and for his support towards fulfilling their objectives and their dreams. Having a famed memory helps of course. How many institute Directors can remember every staff member, as well as their plans and families?

High-flying individuals such as Marcel Tanner build up an un-paralleled network of contacts, institutions and opportunities, which in turn leads to solutions in so many circumstances. Finally, a most important condition for a "yes by default" strategy is the ability to find constantly new financial resources – something staff of the Swiss TPH affectionately called "Marcel's white rabbit" farm. Most of us were repeatedly amazed about his ability of pulling up finances from the giant virtual hat in his office. Sources of financing that nobody knew about, highly diverse (a good predictor for success in the financial world), and not seldom amazing.

At an institutional level, his political astuteness was consistently a model for us to follow. Support by the local and national governments increased markedly over the 2 decades of his Directorship. The one moment that stands out in my mind was during one of the many currency shocks when the Euro lost massively in value against the Swiss francs, and many of our European grants lost 10-20% of their value. This situation similarly affected Swiss industry, and the federal government created an exchange rate support mechanism to help them overcome the crisis. How many other academic institute directors went during this time to see the Federal Minister of Finance, arguing that a research institute getting European grants was an export industry similar to watch makers or machine manufacturers, and therefore deserved support? The substantial amount received from this compensation mechanism by the Swiss TPH is a remarkable tribute to Marcel Tanner's savvy in managing the science business.

Marcel's positive outlook, his pro-active and highly skilled managerial approach combined with high scientific standards are unique and have massively shaped the Swiss TPH under his leadership. It has also led many of us to become better scientific managers, an attribute

often downplayed by conventional academics. There is undoubtedly nobility and long-term success in managing affairs well, while striving to establish a better world. This is the legacy for which I am particularly grateful to Marcel.

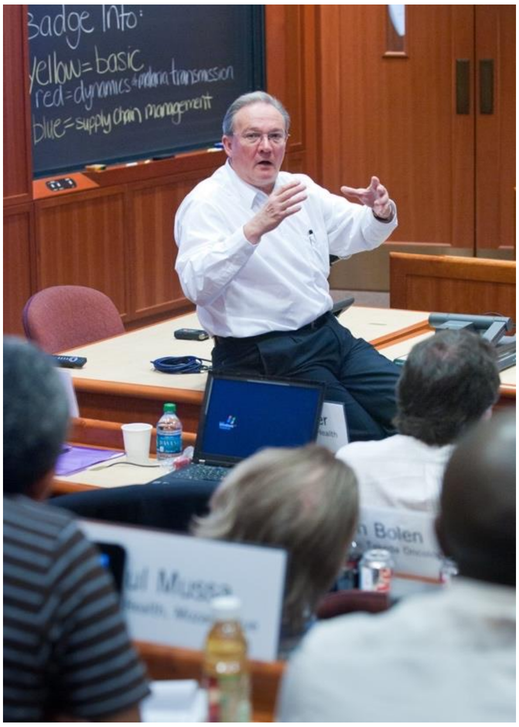

Figure 1. Marcel Tanner teaching at Harvard University.

In honour of Marcel Tanner: Long-lasting impact

Frederick Masanja[1]

[1] Ifakara Health Institute (IHI), Tanzania; fmasanja@ihi.or.tz

I first came to know Marcel Tanner in the year 1993 when I was employed at the Dar es Salaam Urban Health Project (DUHP), under the Dar es Salaam City Council, a project that was funded by the Swiss Agency for Development and Cooperation (SDC) and was executed by that time Swiss Tropical Institute (STI). The project aimed at strengthening Health Systems and had placed emphasis on delivering improved services at the primary level with increased community participation. Marcel used to come for the Board meetings and that is when we came to know each other though not a very close level. But since then, there has been numerous and memorable and very exciting experiences with him. He later came to know that I was the younger brother of Honorati Masanja who at that time was working at Ifakara Centre as Data Manager. I think from there, we became more closer in our relationship. In 1998 I had the opportunity to visit Basel for the first time for capacity building and also to get to know the people I was working with at the DUHP project. I remember I was brought to meet Marcel in his office and we had some time to discuss issues around the importance of the project and the overall progress of the project work, and my work. We didn't have much acquaintances apart from normal talks around work related matters. I left DUHP in March 2000 after I was linked to SDC by the Project Manager by then, (the late Pierre Pichette) and I applied for the position of Administrator at SDC (Swiss Agency for Development and Cooperation)/ Embassy of Switzerland. I joined SDC officially in May 2000. Here again I met with Marcel Tanner and he was very happy to see me working for the Swiss Embassy and encouraged me to work even harder. I remember at two-three different times he invited me to join him and some of his colleagues at the Swiss Garden hotel in the evening just for talks outside work while enjoying his drinks and work! (Responding to emails or working on other documents). I remember in 2004 and 2006, I had the opportunity to visit SDC Headquarters in Berne for IT basic training. So, after the training I travelled to Basel to visit my brother Honorati who was doing his PhD. Again, here I had the chance to meet Marcel though not on an official note but he always warmly welcomed me each time I visit his office and he would bring to introduce me to some other colleagues at Swiss TPH and to those who already knew me since the time in DUHP. One of the most interesting incidences with Marcel happened in the year 2009 when he visited the Embassy offices at Kinondoni Road. I remember we met on the corridor and he asked me after greeting each other and joked a bit (as his norm) before saying 'Frederick, I am looking for someone like you' I looked into his eyes and I said, someone like me?... I said what do you mean? Then I remember my boss, Claire-Lise overheard what Marcel was asking me and she shouted on top of her voice from her office, 'He is not going anywhere! Marcel. Then Marcel squeezed my hand and said, come at the Swiss Garden after work and we can talk more. I said Okay. Claire-Lise came from her office and said to Marcel if you are looking for another Frederick that's fine but he is here to stay. We all laughed! So, that evening I went to meet him at the Swiss Garden Hotel and he said to me we are looking for someone to fill the position of grants manager or senior administrator and he asked me to contact Dr. Hassan Mshinda. I managed to find someone and luckily, she did fit the requirements and I was so happy about that. At the end of the year 2010 I decided to resign from SDC and I remember to have told Marcel about my decision. He was not very happy with my decision saying that it was too soon to leave without having found another job. I explained to him the reasons and he agreed and he said he would help me but did not guarantee to have something soon, I said it is fine with me. So, I stayed home for some 5-6 months before Marcel informed me sometime in July 2011 to go and see Peter

Balzer, the Chief Operations at Ifakara Health Institute for a job at one of the projects to work as administrator. At the end of the interview Peter Balzer told me that you have done very well Frederick…so, what do you think I should tell Marcel? I laughed and said, If I have done very well, tell him when do you think you are going hire me? He laughed and said Okay. I was called while driving back home that I should report the next day! After my probation in September 2011, I was confirmed for the position but it lasted only for nine months when I was appointed by the IHI Directorate to be the branch manager for Ifakara branch from July 2012. I was surprised that few months later Marcel Tanner came to Tanzania and visited Ifakara with a delegation from Switzerland. He did not hide his feelings during the party in the evening at the poolside when he held me by the hand and showering me with praises as the 'Mkurugenzi' for Ifakara branch. He explained a story that before there were 'rats' who were eating the boat and wanted the boat to capsize but now, we are in good hands and he said, "sasa tunaenda pamoja katika mtumbwi mmoja" meaning "now we are sailing in one boat'.. It was a memorable day for me amongst many such days during his numerous visits with several missions and friends to Ifakara. The canoe trip along the Kilombero river is a culture that I cannot forget…in the beginning I was afraid but he kept urging me that it is fun…finally I became used to these trips because of the enjoyment of stories about the crocodiles and hippos in the river Kilombero. The evenings after the canoe trip were always very vibrant with 'nyama choma' mainly 'ngiri' of course with a variety of other snacks and drinks while listening to Marcels stories about the genesis of Ifakara and other institutions around. The hunting stories are the most enjoyable part of evening stories after the tour of the Ifakara institutions and a bit of work during the day…this is where Marcel takes the stage with all sorts of jokes while enjoying his wine and in the late hours he changes to whisky (he brings special bottles with him) and cigar smoking competition without dropping the ashes from the cigar! Towards the end of 2012 beginning of December, Marcel came again to Ifakara and this time he came with a brochure from Swiss TPH and called me in his room at the main house and said to me, read this and apply for this course. I think it will be very useful for you now as a leader of the branch. You need to be equipped with this knowledge so that you can speak the same language with the research scientists. He said, "register for this course and we will pay for you… It will be good for you to get a Master in Business Administration at the same time you get some science injections" I said its fine with me, but I didn't know he wanted me register as soon as possible because the course was starting in March the following year. So, when he came back in January 2013 he asked if I had filled the registration forms and I said not yet, he looked at me with narrowed eyes as if conveying a strong message then he said, this is needed as of yesterday! Let's go in your office and we will fill it together, I have to go back with it. I realized this was serious business! I reported for my semester modules 3 and 4 in August 2013. We had moments together with Marcel during the time and I remember during my first travel back home after the modules was an interesting one but also very tough one. I remember Marcel asked me to go to his office in the evening on the eve of my travel. We went together in his car at the train station SBB-Basel in a chocolate shop and bought me bag full of varieties of chocolates saying bring this to your family and friends and Honorati. I said to him, my suitcase is already full materials from the modules and other things and I have two carry-on luggage. But Marcel insisted that it will be okay once I am in the train and after I check-in at Zurich airport I will be fine. So, I was wondering how am I going to carry this bag with all my other luggage! That early morning had some showers so I had to take a taxi. Sometimes during my MBA modules, he would call Axel Hoffman or Amena Briet to tell them he needs to see me in his office. So, whenever they announce in the class my colleagues especially the ones from Tanzania were wondering about my relationship with Marcel. So, one day I told him that my colleagues from Tanzania want to meet with you and he agreed. We went and he was very happy to learn from them and until now one of them (Dr. Ntuli Kapologwe) are in contact with Marcel for various work-related matters. In another incident, I remember the Swiss

Tropical Institute 70 years celebrations. There were many events that were prepared. I attended an event where there were presentations about the history of Swiss Tropical Institute and how they came to East Africa and especially Ifakara in Morogoro Region to the current Swiss Tropical and Public Health Institute. In one of the events, there was dinner prepared in one of the hotels near restaurant Schützenhaus. Marcel danced with one lady (I cannot remember her), but it was a moment full of joy from everyone around. During the time when I was doing my MBA research it happened that, I took a long time to finish it and Marcel intervened in the situation when he saw that I was almost going to despair. He told me, you have to come and push it from here otherwise you will not finish. I remember I was staying at the student's hostel where his office is on the ground floor and I was on the 3rd floor. So, each time I come down he could see me going out. There was no way I could pass without him seeing me unless he was away on one of his many duty trips. So, one day I asked him jokingly that, did you put me here in this accommodation so you can monitor my movements? and he said while smiling, yes, I have to know your whereabouts!One day we went together for a 'working dinner' with my thesis at hand which he wanted to cross-check my progress. I had to record the comments that he was telling me because we were eating and drinking wine. So, I thought if I just listen, I will forget after a few glasses! I must say that, Marcel had played a big role in my MBA-IHM from the beginning till I finished. He injected whatever I requested from him whether materially or monetary. His moral support during all the years I was doing my MBA is just immeasurable. One day he told me, Frederick, 'ninaona nuru mbele yako' meaning, 'I can see light ahead of you'. You are going to make it.....He always encouraged me and wanted me to relax and not make things look difficult. In June 2018 I came back again to Basel for preparation of my MBA defense. Marcel offered me and my wife Alice accommodation at a friend's house. On the day of defense, it was very tense for me especially knowing the panel that was going to be there. I remember after presentation I was asked some questions and then was sent out. After some minutes I was called back and I was told that I have passed. Tears of joy came out and there was a moment of silence, and I said, 'Thank you so much, you all know how I have struggled to accomplish this thesis. Thereafter, it was followed by an 'apero' and later in the evening Marcel and Suzy invited us at the Tennis Club for dinner. There were also some friends and I remember Prof Don de Savigny and wife, Honorati Masanja, Markus and wife. This is my most memorable time of my life with Marcel. I will never forget in my life! First, I did not know why he did this for me, when I asked him, he said, yes Frederick you deserve this… your study journey was long and difficult and there were times I felt you would end up on the way or you would drop something that I would not have liked to see happening. I am happy that you have done very well. That was not all, before the end of the party he said, I want to hand you over an envelope with train tickets to Ticino through St Gotthard Mountain. He then took the envelope and started directing me how it works to reach there. A hotel was booked for us to stay overnight. The boat trip was fantastic, we went through several touristic areas along a beautiful lake. At the final destination we boarded a train and went through St Gotthard Mountain to Ticino. The trip was very exciting with so many beautiful sceneries all along the way. So, without Marcel I would never have thought or have the slightest dream of visiting such a wonderful place!

The more recent one is when Marcel came to Bagamoyo for the Health systems course, he invited me to go and sleep in Bagamoyo so that we can catch up face-to-face after a long time! Indeed, it was nice to see each other again after two years and as usual he likes to crack some jokes that we understand each other. I have tried to highlight some of the memories that I could remember, but there are so many memorable incidences and experiences with Marcel. I wish him a Happy 70th Birthday and many more years. So basically Prof. Marcel Tanner has impacted not only my career life but also in my own private life and my family as whole. There are so many things that he has done to me that directly helped my career path until today. I sometimes call him my career coach especially in my position as

the branch manager. He always jokes with me that; he doesn't need this MBA bazaar…he says "you just have to ensure you make things happen at the right time and right place".

I take this opportunity to thank him very much for his kind heart. May GOD bless him to blow 100 candles for his birthday.

Mutual learning for change

Hassan Mshinda[1]

[1] Former Director of the Ifakara Health Institute (IHI) and Director General of the Tanzania Commission for Science and Technology (COSTECH); mshinda@icloud.com

I knew I had my work cut out for me when my peers coerced, ahem, assigned me to pay tribute to my (our) mentor, coach, and good friend, Professor Marcel Tanner, who has clocked seventy (70) this year. The onus was not in the want for words but rather in the lingering fear that the space and time to express my thoughts and feelings about the professor may be inadequate because there is just so much one would want to share. And then there was the need to maintain the element of surprise, my peers had suggested that the professor should not be in the loop that we are about to pay homage to him and since I was at the business end of that "elaborate scheme", I diligently obliged. Bringing these factors into the equation, I found myself worrying for an obnoxious amount of time that I might suck you, the reader, down a rabbit hole and lose you along the way but still, it was with utmost pleasure, nay privilege, that I took up the task – all along hoping to live up to expectations – so here you go. Ask anyone who knows Professor Tanner well and none would hesitate to give testimony to his illustrious career and contribution to academia, nurturing and development of individual scholars (scientists), and building and strengthening of institutions. Needless to say, there is more to the person than meets the eye. His first passion might be teaching, but he is also fascinated by nature and history and he actually introduced me to the nitty-gritty of hunting game in the wild and lectured me on the country's hunting laws. The unassuming professor was also deeply involved in community work in Ifakara during his stay there. I first met the professor in 1984, I was then working at Ilala District Hospital but in the evenings I would volunteer at Muhimbili National Hospital (MNH) in the department of parasitology doing research and professor Tanner was on a recruitment drive at MNH. His recruitment drive was born out of the need to man his staff at Ifakara with trained professionals because at the time he was taking the reins at the institution, then known as the Swiss Tropical Institute Field Laboratory (STIFL), there was a serious shortage of professional staff. The story is that due to a shortage of staff, Professor Tanner was prompted to take up other roles such as dabbling in the maintenance of the office generator and swimming pool and his wife helped him in his other role as an accountant and cashier. He was also known to be time-sensitive, earning him the moniker "mzee wa saa", one of many, because he had to multi-task, just to maintain a semblance of normalcy at the workplace, time was always of the essence. Back to my first encounter with Professor Tanner. It took place at the Department of Parasitology, by then the Faculty of Medicine at Muhimbili, where he was looking for medical technologists to work at STIFL and I was introduced to him by my tutor, Mr. Ramji, who was the Chief Technologist at the department, and the Professor expressed interest in recruiting me. It should be noted that my ambition at the time was to pursue further studies in Biochemistry and not work for STIFL but the Professor managed to convince me to study parasitology instead, his reasoning being that more experts were needed in that field in the country, and I agreed to join Ifakara only after completion of my Diploma studies. A few months after I started my studies he continued to send me publications and reports from Ifakara to update me on the progress of the institute and its work and we remained in contact until the arrival of Dr. Don de Savigny, who took over the Directorship of STIFL and I joined the team after my studies.

It is important here to understand that it was the Professor's persistent communication style that made me develop more interest to accept the offer to work for the institute. And thus began a new chapter in my career, the development of which owes much to Professor Tanner, and in the course of the years that we have come to know each other, I can bear witness to the Professor's Midas touch.

1. **Tenure at Ifakara**

Professor Tanner's tenure lasted for four years, from 1981 to 1984. As stated earlier, it was not a walk in the park because when he first arrived at Ifakara there was a shortage of trained staff and he thus had to keep tabs on all the institute's inventory and perform all sorts of chores. Furthermore, he faced other unforeseen challenges and had to put with Tanzanian employees for several days where he came to learn firsthand that they were extremely sharp and needed more training to develop. On top of that, part of his job was to lecture at the then Medical Assistant Training Centre (MATC), now the Tanzania Centre for International Health. However, the most urgent challenge he faced was the recruitment of trained technologists who would be willing to leave city life and work in a peripheral location such as Ifakara. His philosophy of recruiting and developing Tanzanian scientists has been a guiding principle in leading the institute, a principle rooted in the history of cooperation between Tanzania and Switzerland which dates back to 1966. On October 23, 1966, Tanzania signed its first agreement with Switzerland as a country on technical and scientific cooperation, which in essence was not development aid but rather focused on teaching and training schemes. The agreement was signed years after STIFL had already been established and was operating in the country. The cooperation agreement put emphasis on the need to translate action for the benefit of the people without looking at how much one party contributes so long as that party has had their contribution, the most important thing is that they seek knowledge together to make a difference. It was in the spirit of this philosophy, which he readily admits to having borrowed ideas from and adjusted to relate and meet the needs of the time, that informed his decision to seek Tanzanians to recruit at the laboratory in Ifakara and I just so happened to be one of them. Unbeknownst to many scientists then, Ifakara as an institute was located in a remote area but the funds for training scientists outside the country were available, and Professor Tanner was adamant to hire Tanzanian scientists because, among other things, he wanted to build a research ecosystem and not just a research centre at Ifakara.

2. **District-based research centre**

The location of the institute was chosen on the basis that it had to be a district-based research centre because it is mainly at the district level where policies are translated into action, and at the district level where you are closer to the community, understanding the communities better than somebody who is sitting at the national level. Given its peripheral location, Ifakara was at an advantage as a research and resource centre compared to other institutions which were based in major towns and that gave the institute its competitive edge. Even in its mission statement, throughout Ifakara maintains that it is a district-based research and resource centre – it looks at problems and their solutions from a district angle. The philosophy to make Ifakara a district-based research and resource centre came at an opportune time because this was when Tanzania was implementing decentralization by devolution and that was translated into Ifakara's mission to have a district focus.

3. Development of Tanzanian scientists

His legacy goes beyond managing Ifakara as an institution. Yet to better understand Professor Tanner's legacy, one must first understand the multifaceted roles he played during his stay in the country as head of Ifakara and after he left the country. He left an indelible impression on the communities he has served and on individual scientists. As a full Professor in the Faculty of Medicine and the Faculty of Sciences, on retirement at the age of 65, he delivered his final lecture which he titled "No Roots, No Fruits". No roots, no fruits: this is a maxim that he religiously advocates and it hinges on the premise that one cannot build strong and effective institutions without investing in capable manpower. It is against that backdrop that he was actively involved in furthering the development of Tanzanian students (scientists) by seeking funds for training and through the Rudolf Geigy Foundation, he managed to assist many scientists to pursue further studies abroad and the first beneficiary was Abraham Mnzava, who went on to become a World Health Organization (WHO) scientist specialized on disease vectors and also worked in various African countries such as Egypt and South Africa. I too was among the beneficiaries of his initiatives along with other notable figures including the late Dr. Mwele Malecela, and this was because he was more interested in building research capacity in the whole country and not confining to one institution. He also helped secure funds from Swiss Agency for Development Cooperation to train NIMR scientists and then Ifakara scientists. His activities while working in the country and after leaving the same offer a glimpse into the Professor's character and convictions. As a Professor, he has been a supervisor of many students pursuing their masters and PhD degrees. After you have designed your study and worked on it, he would go through each chapter and advise you on where to make corrections. He has supervised over 50 Tanzanian PhD students and continues to do so to date.

4. Builder of institutions

To date, he is a firm believer in the principle that institutions have to do research, training, and direct service application because that is the only way one can see how things have translated into action instead of just doing research and writing a publication."You must have dirt on your boots," he would say, alluding to the painful fact that one (as a scientist) has to do more than just desk work to have an impact on communities. It might suffice to state that Professor Tanner has left his footprints on the sands of time because not only has he helped to build a strong institution (STIFL) which steadily grew to become Ifakara Health Institute (IHI), but he has also helped build good rapport and working collaboration with other national institutions such as the Muhimbili National Hospital (MNH) and the National Institute for Medical Research (NIMR), the latter since its formative years.

5. The National Institute for Medical Research (NIMR)

In his early days at the helm of the institute, Professor Tanner took the initiative to seek an audience with Professor Wen Kilama of the National Institute of Medical Research (NIMR) to try and get it through to him that he sees Ifakara as a Tanzanian organization and all that was needed is for the institutions to work together and help to build each other's capacity. Professor Kilama was initially skeptical but later came around because there was a time when Tanzania was facing an acute shortage of foreign currency and after reasoning for some time with Professor Kilama, Professor Tanner came to the rescue of NIMR because at the time STIFL was financed in Swiss francs so instead of changing the francs, he would take Tanzanian shillings from NIMR in exchange for the francs which would, in turn, be used to procure research materials, laboratory agents and other lab equipment.

6. From the Medical Assistant's Training Centre (MATC) to the Tanzanian Centre of International Health

Another experience that I cherish to this day is a product of the tale of the Rural Aid Centre (RAC). The centre and STIFL share a similar history in that they were both the brainchild of Rudolf Geigy, founder of the Swiss Tropical Institute in Basel. The RAC later became known as the Medical Assistant Training Centre (MATC) and was handed over to the Tanzanian government. The centre was established to train frontline health workers and teach parasitology for Muhimbili National Hospital students before the parasitology department was established at MNH, and it was at the centre where Professor Tanner was teaching and I too was introduced to teaching and communication skills at the same centre. It was Professor Tanner who smoothened the handing over of the centre to the government and became part and parcel of the Ministry of Health, but unfortunately, after it was handed over it started underperforming because of lack of funds and its infrastructure began to dilapidate. The state of affairs at MATC after it was handed over to the government served as an eye-opener because it was realized that the same fate could become of Ifakara once it is wholly handed over to the government.

7. From the Swiss Tropical Institute Field Laboratory (STIFL) to the Ifakara Health Institute (IHI)

Working on the principle of research, training, and service support, he set the bar so high and laid the foundation for the transformation of STIFL to IHI, and thus becoming the poster child of excellence that it is today. During his tenure, he made sure that STIFL does not remain a Swiss island because he was more concerned about building a national research ecosystem and not just Ifakara as an institution. His vision was not to just open up Ifakara for Northern institutions as a field site but rather sought funds to support Tanzanian scientists and institutions and create partnerships for Northern research centres to work with Southern institutions. In the case of STIFL, there were fears that if it were wholly handed over to the government it would lose its autonomy and follow the route of MATC, the poor performance of MATC was a good lesson when it came to deliberating about the fate of Ifakara.We, therefore, worked together and came up with a structure that would allow Ifakara to remain independent but with the involvement of the government in decision-making together with the Swiss government and the Basel Foundation. Basel Foundation owned the infrastructure that Ifakara was operating on and the foundation was more than willing to donate the building to what became known as Ifakara Health Research Development Trust, and I worked closely with Professor Tanner to establish the trust.

Initially, STIFL was renamed Ifakara Centre' and made an affiliate of NIMR, but it was later made into a trust and renamed, 'Ifakara Health Research and Development Centre (IHRDC) before it became known as Ifakara Health Institute. At the establishment of the trust, there were two major issues of concern, first, we wanted to maintain the partnership between the founding members, and secondly, we wanted to diversify our sources of funding, that is, instead of depending on funding from Switzerland, we decided to also seek funds from elsewhere, an approach that I learned from Professor Tanner. Truth be told, this is how I came to learn about the flower concept – a concept that stresses the need for projects that are taken up by the institution to not only be implemented but also contribute to the core (overall running costs of the organization) – and it was not clear to many research institutions in the country because when working with the government, it is the state that supports the core by paying salaries, utilities and so forth but at Ifakara it was quite the opposite.

8. The flower concept

Of course, at Ifakara we had the support of the Swiss Agency for Development Cooperation, but we still had to seek funds to pay salaries, pay utility bills and competitively find money to do research. The flower concept is basically the notion that through partnerships you attract projects and these projects meet their cost but also contribute to the core costs of running the organisation. Years later, the flower concept came in handy when I was appointed as Director General of the Commission for Science and Technology (COSTECH). When I was at COSTECH it was the other way around, I never had to seek funds to pay salaries or utility bills, these funds were readily available from the normal budget because the commission is established by an act of parliament. My job was to look for collaborators to finance research institutions in the country while we manage the research projects, it was easy for me because of the experience I had gained from Ifakara and I also had a network of donors and I owe that to Professor Tanner.

9. The risk-taker (from Ifakara to Dar)

As a manager, he always made strategic decisions that involved taking risks and that led to some quarters labeling him as someone with dictatorial tendencies, but true to form, when he was convinced of his vision, regardless of what anyone would think of him, he would go ahead and pursue it. When I was managing Ifakara, the main challenge was attracting good scientists to come and work at the institute because it was in a remote location. I realized that good scientists prefer to work in the city as consultants instead of doing research in the field. I also came to learn that there was a shortage of consultants in the country and so I decided to seek funds to establish a consultancy firm in Dar es Salaam as part of Ifakara, and I did secure funds from the Swiss Agency for Development Cooperation, but I was not prepared to rent an office, I wanted to purchase a building. The idea was to have another source for funds by owning a building and letting space to other tenants – the thinking at the time, collecting rent from tenants for a whole year would be a substantial amount of revenue. I thus sought advice from Professor Tanner and he was prepared to support me to purchase a building in Dar es Salaam from the get-go, and I went ahead did the needful and to this day it is the same building that houses Ifakara's offices in Dar es Salaam. This was a very risky move because we took the money on the condition that we will pay it back through the rent we would be collecting to the equivalent of the grant we received, we took the risk and it paid off. In another instance, he was also once prepared to advance funds for research before they were released – in normal financial management, that was not proper – but as a leader, he would take decisions that were risky, and yet he was always confident that the decisions would be fruitful. His approach was to run the team, work with and through the team but sometimes take the leadership role when and where it was necessary to show the way which at times was misconstrued for him being a dictator at the workplace, and yet this very same person was always ready and willing to discuss and explain the basis of his decisions.

10. From Ifakara to Bagamoyo

The story of how IHI opened offices in Bagamoyo is inextricably entwined to the impact the institute has had on communities and the direct and indirect intervention of Professor Tanner through his vast network. He offered us support from behind the scenes when he started constructing the Bagamoyo vaccine research centre, but I will be doing a great injustice if I leave out the impact of Ifakara on communities before getting into details of how the Bagamoyo office came to be. Through the treated bed nets project, we managed to reduce malaria by 27% among children, and anemia and malaria infection by 50%, a feat that led to the formation of a national programme. Furthermore, new approaches in malaria

prevention such as that of using malaria drugs for children when they came for immunization were first demonstrated in Ifakara and later validated in other countries and now it is a strategy that is promoted by the World Health Organization (WHO). Another feat was the intermittent preventive treatment in infants (IPTi) which was developed and tested in Ifakara before being adopted by WHO. Social marketing of insecticide-treated bed nets also started in Ifakara and then it was promoted to become a national programme and even stimulated the establishment of manufacturing facilities in the country such as the A to Z factory which was visited by then US President George W. Bush when he was in the country. As a result of these achievements and Professor Tanner's vast network, we received visitors from Geigy Foundation and GSK who were looking for a site to conduct malaria vaccine trials, and because of our work in Ifakara, they were satisfied that we had enough experience to work with them in doing clinical trials. The problem was that at the time malaria incidences in Ifakara have been reduced to such an extent that it was not adequate to conduct proper trials and so they left and encouraged us to find another place and that's how we moved to Bagamoyo; where we were setting-up modern clinical trial facilities.

11. After Ifakara

After Professor Tanner returned to Switzerland, he revived the scientific cooperation agreement between the two countries, which was crucial because at one point in the 2000s, the then Swiss Secretary of Education, Research and Innovation visited Tanzania and went to Ifakara. By reviving the agreement, he also managed to secure additional funding for scientific cooperation which entailed funds for training scientists and working in collaboration in conducting scientific research. He remained supportive of what we were doing even after he joined the Swiss Tropical Institute as the head of a department, before becoming the head of the institute itself. One incident remains clear in my memory, after finishing my Masters' Degree I was interested in doing molecular biology and the Professor was keen to know what I was going to do next after my studies and at that time we neither had the equipment nor materials to conduct research in that field. On that background, Professor Tanner volunteered to bring to Ifakara a PCR machine along with a Swiss scientist with whom I worked. We borrowed the machine first to see if it would work in our environment, after a while he took it back but later returned it after we requested the same. Mind you, at that time the Swiss Tropical Institute did not have such a machine of its own, it was borrowed from another organization, but Professor Tanner agreed to give us priority over his own institute, a gesture that I shall remain grateful for. It was through his support that I was able to start questioning why there is anti-malaria drug resistance and we started working on the genetics of the malaria drug resistance while all along he was supportive in ensuring that there was a strong partnership between Switzerland and us. We were able to establish a molecular lab for malaria drug resistance at Ifakara and we received samples from as far as Gambia, Malawi, Kenya and Uganda which were analyzed at Ifakara.

He made sure that we had the necessary facilities and technical support to develop our line of work and we could also look for other resources to further the line of work and eventually we became the first molecular biology laboratory in public health in the country.

12. Community work

During his stay in Ifakara, Professor Tanner went out of his way to actively engage in community work that was beyond his call of duty. He introduced four programs, two of which were community-based and two were clinical-based. The hospital-based programs mainly focused on interventions for major liver diseases and malaria drug resistance among residents of the area. Among the community-based programs with far-reaching impact initi-

ated by Professor Tanner is one at Kikwakila village in Ifakara which focused on the interaction between nutrition, infection, and immunity after he conducted research on the same. The Kilombero Health Research Programme (KIHERE) established projects to study the transmission and control of schistosomiasis, established Health System Research in Mlimba division, and supported Kilombero district in implementing National Primary Health Program. This also led the Professor to promote primary health care at the grassroots which included advocating for more training for village health workers but recommended that the training should be conducted at the district level near the communities they serve. He was always at pains that when village health workers were sent to train for two years in major towns like Dar es Salaam or Morogoro, they normally never wanted to return to their posts in the villages thus the need to train them closer to their work station, a model that was adopted by the World Health Organization (WHO) in 2008 when promoting primary health care in remote areas, and the Professor had initiated the same way back in 1982 and 1983.

13. "Duka la maombi"

On a casual note, Professor Tanner has not only been a good Samaritan to the scientific community in the country, he is not the kind of person to interact with just the elite of society, he mingles with people from all works of life and his generosity has somewhat earned him a celebrity status of sorts in Ifakara. Whenever he would pay a visit, which he would regularly do to show support for the communities, especially for the Kilombero Health Research Programme (KIHERE) project which he initiated while at the helm of the institute, a horde of residents would gather around to seek audience with him. We often joked that he should open a "duka la maombi", you know, something like a shop to receive requests from residents ranging. People would want to see him for various reasons, some just to learn how he is doing, others to seek counsel, some to ask for a favour or two, and others would go as far as to ask for money to add another wife.

14. The nature lover

Over the years I also came to learn about the Professor's passion for the outdoors. We recently met in Bagamoyo, I did not give away the fact I was assigned to piece together a narrative paying homage to him, where he confided that the one thing he misses the most is the "bush" life. Towards the end of his tenure in Tanzania as the head of STIFL, in September 1984, he set out with some colleagues to walk in the Selous Game Reserve for eight days, the group was made up of eight Tanzanians and eight foreigners, along with his wife. The Professor had already decided in 1982 that he would walk in the Selous after reading the diary of Imperial German Army General Paul von Lettow-Vorbeck, which he says is utterly boring but offers a good description of nature, and Peter Matthiessen's book, Sand Rivers and the diary of Brian Nicholson, a former warden at Selous Game Reserve. Using information dug out from the book and the diaries, he charted an area where he wanted to walk, and they boarded a plane from Ifakara. Bringing along three guns and ammunition, two game scouts, and enough supplies for eight days. They would walk from sunrise to midday and then set up camp, they trekked from Lukulu to Mkangira and hunted when they set camp and they managed to shoot down some game, including a big gnu. He gave a word of advice about taking a trip in the wild: Always take along someone who is afraid because that person will always be vigilant out of fear and in the process he will be unwittingly keeping watch of any lurking danger while the rest sleep soundly.

15. Final word

To say that Professor Tanner started major transformations at the institute that made it what it is today might be an understatement. That is not to mention the number of students (scientists) he helped develop their careers through funding from sources from without to finance their studies. He tirelessly sought funds to not only strengthen STIFL but also advance its manpower and that of other institutions without prejudice. He had also put in place structures and inculcated a culture of teamwork, multi-tasking, and mutual learning that he bequeathed to his successors who built upon the same, which has been crucial for operational purity of the institute and the purpose it was meant to serve. His continued dedication and support to both institutions and communities he interacted with and individuals speaks volumes about the Professor's character and his passion to help people from all walks of life.

Professor Marcel Tanner, we salute you. Happy Birthday!

Marcel Tanner – a partner and friend

Max Price[1]

[1] Former Vice-chancellor of the University of Cape Town, South Africa; max.price@uct.ac.za

I first met Marcel in 1986 at the London School of Hygiene and Tropical Medicine. He was studying on the MSc Community Health in which I taught a module. He was quite taken by a teaching tool that I had computerised to illustrate the consequences of health resource allocation decisions at district level. He invited me to spend some time in Basel at the then Swiss Tropical Institute to run the teaching exercise with his students and develop the model further with one of his PhD students, which I did the following year. We used the data he had collected during his long period of fieldwork at Ifakara, Tanzania, to develop a realistic model of an actual district health system (the Kilombera district). This laid the foundation for a long, though long-distance, relationship in which Marcel kept popping up holding one or other senior position in any number of organisations, and each time drawing on our connection to further some greater goal in research, public health, capacity development, and partnerships. In the 1996, I became dean of Health Sciences at Wits University and in 1997 Marcel became Director of STI. We would exchanged views on health research priorities and the teaching of public health and managing complex institutions. Marcel became co-chair Swiss Commission for Research Partnership with Developing Countries and his personal interest and determination to promote such partnerships was always evident in his energy and enthusiasm and personal networks that he nurtured with researchers in developing countries. When I was Vice-Chancellor of the University of Cape Town, our regular dinners acquired a much classier venue, viz. the home of the Swiss Consulate General in Cape Town, since he visited regularly as part of the Swiss government's scientific delegation to further partnerships with South African researchers. He facilitated the establishment of joint chairs in environmental health between UCT and the Swiss Tropical and Public Health Institute. In his capacity as co-principal investigator with Novartis and Medicines for Malaria Venture (MMV) focussing on drug discovery for malaria based on novel drug targets, the Drug Discovery and Development Centre at UCT, under Prof Kelly Chibale, became a key partner in these global research efforts. And then, as I moved into in semi-retirement, (a concept Marcel will probably never know even though he is a few years older than me) I unexpectedly had the opportunity to work much more closely with him when I was appointed a member of the Expert Commission, Fondation Botnar. Marcel was co-chair of the Expert Commission. His vast experience on committees and commissions was now evidenced in his impatience with bureaucracy, his irritation with poorly written research proposals, his ability to cut through to the essence of a proposal, and his acquired imperiousness, no doubt the result of the many positions of authority he has successfully occupied over the years. When I recall the encounters I have enjoyed with Marcel over three-and-a-half decades, each of them felt like a reunion. With little contact in between, they were nevertheless as if we were close buddies, sharing deep mutual respect, bear hugs conveying genuine warmth, and a shared sense of the bigger project of global health. I enjoyed every one of them.

Thank you Marcel for your loyal friendship.

Marcel Tanner – his vision for institutional parallel pandemic preparedness

Nicole Probst-Hensch[1]

[1] Swiss Tropical and Public Health-Institute (Swiss TPH), Switzerland; nicole.probst@swisstph.ch

Marcel Tanner is an archetype of a global public health expert and strategist. Long before the occurrence of the COVID-19 pandemic he had to vision to integrate the Swiss Tropical Institute with its main focus on infections and neglected tropical diseases and the Basel Institute of Social and Preventive Medicine with its main focus on environment and non-communicable diseases. His deep understanding of the complexity of health and wellbeing, his ability to build, implement and maintain long-term partnerships, and his motivating personality to engage people for his mission transformed Swiss TPH into the largest Swiss Public Health research institute well prepared to address local, national and global public health threats with the breadth and interdisciplinary expertise needed. The COVID-19 pandemic underlines the relevance of Marcel Tanners strategy and vision. It has broad and in part detrimental direct and indirect consequences for global public health. In addition, the fast spreading virus has brought to light parallel pandemics and public health threats and their complex interdependencies. From a public health perspective this is a "beneficial" consequence of the COVID-19 pandemic. First, the COVID-19 pandemic is proof-of-principle for the concept of One Health. The epidemic rates of biodiversity loss, land use fragmentation, and urbanization increase the risk of new zoonosis, which can then easily spread in a world of globalized trade and travel. COVID-19 thus points to the relevance of transboundary efforts in maintaining planetary health by all means. Second, COVID-19 demonstrates the global relevance of the dual disease burden consisting of both, infections and non-communicable diseases (NCDs). Infections remain an important public health threat in all countries. Demographic aging which comes with higher rates of NCDs and infllammaging adversely affects susceptibility to infections. High income countries must learn to not ignore the threat of infections, while low and middle income countries must learn to integrate NCDs into their disease control efforts. As both infections and NCDs are severely affected by environmental pollutants all countries in the world must work jointly towards maintaining and improving planetary health. Third, the pandemic of mental disorders could no longer remain hidden in COVID-19 times. Citizens fear of infections combined with the adverse economic and social effects of the pandemic containment measures led to stark increases in mental health symptoms. In most cases treatment capacity was inadequate to meet the increased demand for psychological and psychiatric treatment. The goal must be that stigmatization of mental disorders and underfunding of mental care is addressed by societies and health care systems in the future. Fourth, the pandemic has affected the poorest citizen groups the most and has therefore widened the social inequity gap. All parallel pandemics, ranging from unhealthy lifestyles, to unhealthy environments, to NCDs and mental diseases and finally to poor access to quality care including vaccinations are exhibiting social inequity gaps. Social equity is therefore a priority consideration of all public health activities ranging from research to policies.

.

Funding: "This research received no external funding"

Conflicts of Interest: "The authors declare no conflict of interest."
